# Supplementary material for: Music and dance in respiratory disease management in Uganda: a qualitative study of patient and healthcare professional perspectives
Source: BMJ Open. 2021 Sep 23;11(9):e053189. doi: 10.1136/bmjopen-2021-053189 (PMC8461694; doi:10.1136/bmjopen-2021-053189)
Supplement: Supplementary data [file bmjopen-2021-053189supp002.pdf]

Kupumua Structured Observation Sheet 1

Trial session:

Date:

Observer:

Location:

People present:

Observation of an active session (could be singing, dancing, PR or PR plus music/dance)

Observation	Session type:
1. Body language	
2. Facial expressions	
3. Speech/expression	
4. Interactions between peers	
5. Interactions with staff	
6. Physical involvement with music, singing, dancing	
7. Disease related behaviour (short of breath, coughing, fatigue, resting periods,	
8. Role within the group. Passive/active. Lead/follow.	
9. Reflexive researcher responses	

19/MAR/2019 Version 1

Philip KEJ, *et al. BMJ Open* 2021; 11:e053189. doi: 10.1136/bmjopen-2021-053189
