# Supplementary material for: Music and dance in respiratory disease management in Uganda: a qualitative study of patient and healthcare professional perspectives
Source: BMJ Open. 2021 Sep 23;11(9):e053189. doi: 10.1136/bmjopen-2021-053189 (PMC8461694; doi:10.1136/bmjopen-2021-053189)
Supplement: Supplementary data [file bmjopen-2021-053189supp003.pdf]

Supplementary Materials

Preparatory Reference Materials

- Does pulmonary rehabilitation alter patients' experiences of living with chronic respiratory disease? A qualitative study Int J Chron Obstruct Pulmon Dis. 2018; 13: 2375–2385. doi: 10.2147/COPD.S165623 https://www.ncbi.nlm.nih.gov/pmc/articles/PMC6087019/
- A development study of pulmonary rehabilitation for patients with chronic lung disease in Uganda https://erj.ersjournals.com/content/48/suppl_60/PA858.abstract
- A qualitative study on the development of pulmonary rehabilitation for patients with chronic lung disease in Kampala, Uganda https://erj.ersjournals.com/content/48/suppl_60/PA3964.abstract
- International research and guidelines on post-tuberculosis chronic lung disorders: a systematic scoping review https://gh.bmj.com/content/3/4/e000745.abstract
- A pre–post intervention study of pulmonary rehabilitation for adults with post-tuberculosis lung disease in Uganda https://www.ncbi.nlm.nih.gov/pmc/articles/PMC5729823/
- Chronic Respiratory Symptoms and Lung Abnormalities Among People With a History of Tuberculosis in Uganda: A National Survey https://academic.oup.com/cid/advance-article-abstract/doi/10.1093/cid/ciy795/5099459
- Beliefs and behaviours towards chronic lung disease - a mixed-method FRESH AIR study https://erj.ersjournals.com/content/50/suppl_61/PA3891.abstract
- Late Breaking Abstract - Health economic burden of asthma/COPD in Uganda, Vietnam, Kyrgyzstan and Greece: FRESH AIR results https://erj.ersjournals.com/content/50/suppl_61/OA2911.abstract
- The silent socioeconomic impact of COPD/asthma in Africa, Asia and Europe – a FRESH AIR study https://erj.ersjournals.com/content/52/suppl_62/PA4215.abstract
- Critical implementation factors to lung-interventions in low-resource-settings – a FRESH AIR systematic review https://erj.ersjournals.com/content/52/suppl_62/PA4214.abstract
