# Supplementary material for: Music and dance in respiratory disease management in Uganda: a qualitative study of patient and healthcare professional perspectives
Source: BMJ Open. 2021 Sep 23;11(9):e053189. doi: 10.1136/bmjopen-2021-053189 (PMC8461694; doi:10.1136/bmjopen-2021-053189)
Supplement: Author's manuscript [file bmjopen-2021-053189.draft_revisions.pdf]

BMJ Open is committed to open peer review. As part of this commitment we make the peer review history of every article we publish publicly available.

When an article is published we post the peer reviewers' comments and the authors' responses online. We also post the versions of the paper that were used during peer review. These are the versions that the peer review comments apply to.

The versions of the paper that follow are the versions that were submitted during the peer review process. They are not the versions of record or the final published versions. They should not be cited or distributed as the published version of this manuscript.

BMJ Open is an open access journal and the full, final, typeset and author-corrected version of record of the manuscript is available on our site with no access controls, subscription charges or pay-per-view fees (http://bmjopen.bmj.com).

If you have any questions on BMJ Open's open peer review process please email info.bmjopen@bmj.com

BMJ Open

Music and Dance in respiratory disease management in Uganda: A qualitative study of patient and healthcare professional perspectives

Journal:	*BMJ Open*
Manuscript ID	bmjopen-2021-053189
Article Type:	Original research
Date Submitted by the Author:	06-May-2021

Complete List of Authors:	Philip, Keir; Imperial College London; NIHR Imperial Biomedical Research Centre<br>Cartwright, Lucy L; University of Plymouth<br>Westlake, Debra; University of Plymouth<br>Nyakoojo, Grace; Makerere University Lung Institute<br>Kimuli, Ivan; Makerere University Lung Institute<br>Kirenga, Bruce; Makerere University Lung Institute<br>Brakema, Evelyn A; Leiden University Medical Center, Department of Public Health and Primary Care<br>Orme, Mark; Department of Respiratory Sciences, University of Leicester (United Kingdom); Centre for Exercise and Rehabilitation Science, NIHR Leicester Biomedical Research Centre – Respiratory, University Hospitals of Leicester NHS Trust - Leicester (United Kingdom)<br>Fancourt, Daisy; University College London, Department of Behavioural Science and Health<br>Hopkinson, Nicholas; Imperial College London<br>Jones, Rupert; University of Plymouth, PUPSMD<br>Katagira, Winceslaus; Makerere University Lung Institute

Keywords:	Respiratory infections < THORACIC MEDICINE, Tuberculosis < INFECTIOUS DISEASES, Thoracic medicine < INTERNAL MEDICINE, Rehabilitation medicine < INTERNAL MEDICINE, Chronic airways disease < THORACIC MEDICINE

SCHOLARONE™
Manuscripts

I, the Submitting Author has the right to grant and does grant on behalf of all authors of the Work (as defined in the below author licence), an exclusive licence and/or a non-exclusive licence for contributions from authors who are: i) UK Crown employees; ii) where BMJ has agreed a CC-BY licence shall apply, and/or iii) in accordance with the terms applicable for US Federal Government officers or employees acting as part of their official duties; on a worldwide, perpetual, irrevocable, royalty-free basis to BMJ Publishing Group Ltd ("BMJ") its licensees and where the relevant Journal is co-owned by BMJ to the co-owners of the Journal, to publish the Work in this journal and any other BMJ products and to exploit all rights, as set out in our licence.

The Submitting Author accepts and understands that any supply made under these terms is made by BMJ to the Submitting Author unless you are acting as an employee on behalf of your employer or a postgraduate student of an affiliated institution which is paying any applicable article publishing charge ("APC") for Open Access articles. Where the Submitting Author wishes to make the Work available on an Open Access basis (and intends to pay the relevant APC), the terms of reuse of such Open Access shall be governed by a Creative Commons licence – details of these licences and which Creative Commons licence will apply to this Work are set out in our licence referred to above.

Other than as permitted in any relevant BMJ Author's Self Archiving Policies, I confirm this Work has not been accepted for publication elsewhere, is not being considered for publication elsewhere and does not duplicate material already published. I confirm all authors consent to publication of this Work and authorise the granting of this licence.

For peer review only

Music and Dance in respiratory disease management in Uganda: A qualitative study of patient and healthcare professional perspectives

For peer review only

Authors: Keir EJ Philip[1,2*], Lucy L Cartwright[3], Debra Westlake[3], Grace Nyakoojo[4], Ivan Kimuli[4], Bruce Kirenga[4], Evelyn A Brakema[5], Mark W Orme[6], Daisy Fancourt[7], Nicholas S Hopkinson[1], Rupert Jones[3,4], Winceslaus Katagira[4]

Affiliations

1) National Heart and Lung Institute, Imperial College London
2) NIHR Imperial Biomedical Research Centre
3) Faculty of Health, University of Plymouth, Plymouth, United Kingdom
4) Makerere University Lung Institute, Kampala, Uganda
5) Department of Public Health and Primary Care, Leiden University Medical Center, Leiden, the Netherlands
6) Department of Respiratory Sciences, University of Leicester, United Kingdom
7) Department of Behavioural Science and Health, University College London, UK

*Corresponding author:

Dr Keir Philip

Email: k.philip@imperial.ac.uk

Physical address: The Respiratory Muscle Laboratory, Royal Brompton Hospital, Fulham Road, London SW3 6HP, United Kingdom

Main Manuscript word Count: 4909

Funding:

KP was supported by National Institute for Health Research Academic Clinical Fellowship award and the Imperial College Clinician Investigator Scholarship. The funders had no say in the design and conduct of the study; collection, management, analysis, and interpretation of the data; preparation, review, or approval of the manuscript; and decision to submit the manuscript for publication. This publication presents independent research. The views expressed are those of the authors and not necessarily those of the NHS, the NIHR or the Department of Health and Social Care. DF was supported by the Wellcome Trust [205407/Z/16/Z].

The research leading to these results has received support from the EU Research and Innovation program Horizon2020 (H2020 European Research Council) under grant agreement no. 680997, part of the Health, Medical Research and the Challenge of Ageing Horizon 2020 action, and funding from the University of Plymouth Global Challenges Research Fund (GCRF) internal fund.

Acknowledgements

We would like to thank the study participants for their time and effort, Stuart Blatston at the 625 for supporting logistical requirements of the project, and Francis Mutesasira for delivering the singing sessions.

Competing interests:

Rupert Jones declares grants unrelated to this study from Astra Zeneca, Glaxo Smith Kline and Novartis and personal fees for consultancy, speakers fees or travel support from Astra Zeneca, Boehringer Ingelheim, Glaxo Smith Kline, Novartis, Nutricia and OPRI

No other authors declare conflicts.

Data availability

All data relevant to the study are included in the article or uploaded as supplementary information. No additional data available.

Contributorship statement:

All authors meet the criteria for authorship as recommended by the International Committee of Medical Journal Editors. KEJP, RJ, WK, and BK had the original idea for the study. All authors were involved in designing the study. DW provided guidance on qualitative methods including design and analysis. KEJP, LLC, GN conducted the interviews. KEJP, LLC, DW, GN, RJ and WK conduced the analysis, which was then discussed with the other authors and refined. The first draft of the manuscript was written by KP. All authors read, contributed to, and agreed on the final manuscript draft.

For peer review only

Abstract: (246 words)

Introduction:

Music and dance are increasingly used as adjunctive arts-in-health interventions in high-income settings, with a growing body of research suggesting biopsychosocial benefits. Such low-cost, low-resource interventions may have application in low-resource settings such as Uganda. However, research on perceptions of patients and healthcare professionals regarding such approaches is lacking.

Methods

We delivered taster music and dance for chronic respiratory disease (CRD) sessions to patients and healthcare professionals. We then conducted an exploratory qualitative study, using thematic analysis of semi-structured interviews with the healthcare professionals and patients regarding i) the role of music and dance in Ugandan life and ii) the perceived acceptability and feasibility of using music and dance in CRD management in Uganda.

Results

Eleven patients with long-term respiratory conditions and eight healthcare professionals were interviewed after selection by purposeful convenience sampling. Four key themes were identified from (interview) analysis: Music and dance: 1) were central components of daily life; 2) had an established role supporting health and wellbeing; 3) had strong therapeutic potential in respiratory disease management; 4) the importance of modulating demographic considerations of culture and religion, and age.

Conclusion

Music and dance are central to life in Uganda, with established roles supporting health and wellbeing. These roles could be built on in the development of music and dance interventions as adjuncts to established components of CRD disease management like pulmonary rehabilitation. Through consideration of key contextual factors, and co-development and adaptation of interventions, such approaches are likely to be well received.

Strengths and limitations of this study

- This is the first study to explore patient and healthcare perspectives regarding the use of music and dance in respiratory disease management in Uganda.
- Using in-depth interviews, triangulated with structured observations and key documentation, enabled a detailed, highly contextualised exploration of themes.
- Purposeful convenience sampling ensured appropriate representation from relevant stakeholders.
- As a single site study, the transferability of findings cannot be ascertained

For peer review only

- The COVID-19 pandemic, and related restrictions regarding group activities including singing and exercise, may have influenced perspectives on participating in singing and dance activities.

For peer review only

Introduction

Chronic Respiratory Diseases (CRD) such as post-tuberculosis lung disease (PTBLD), asthma, and chronic obstructive pulmonary disease (COPD) are leading causes of morbidity and mortality globally[1]. The burden of respiratory disease disproportionately affects people in low and middle-income countries, where over 90% of global respiratory deaths occur[1-3], and is predominantly caused by smoking, respiratory infections, biomass smoke exposure, poor nutrition and air pollution[2]. Prevalence data are limited from Africa[4], however in Uganda specifically, research suggests that CRD are common[5 6]. People with CRD in Africa suffer from a high burden of symptoms amplified by social isolation, economic disadvantage and stigmatisation[7] related to their symptoms. Physical exercise training and self-management education are important components of CRD management, with the interaction between symptoms, inactivity, and psychological impairment key factors in the 'cycle of decline' see Jones et al (2018)[7]. There is interest in developing locally adapted, low-cost high-impact interventions in this patient group, for example a recent programme development study has shown that pulmonary rehabilitation (PR) is feasible and improves quality of life and exercise capacity in people with PTBLD in Uganda[8].

Singing and dance have become increasingly popular adjuncts to conventional disease management strategies for people with long-term respiratory conditions in the UK[9] and other high-income countries[10]. Existing research suggests participants experience a range of biopsychosocial benefits including those related to physical performance, mental health and wellbeing, and social isolation[9 11-14]. Music as distractive auditory stimuli during exercise training for people with CRD can reduce breathlessness and increase exercise capacity[15]. Although a large and growing body of research supports using the arts to support health and wellbeing[16], research in low resource settings is limited. Additionally, co-production of arts-in-health activities is widely appreciated as central to the successful development and adaptation of interventions[17]. Through the engagement of key stakeholders, including staff, patients and family members, such activities have the potential to utilise pre-existing sociocultural resources and minimise dependence on additional external funding or resources.

Data from this study examined the perspective of people with long-term respiratory conditions and respiratory healthcare professionals in Uganda to answer the following questions:

- What are the current roles of music and dance in general life?
- Would the use of music and/or dance in the management of long-term respiratory conditions be acceptable and feasible?

Answering these questions is important to establish if such approaches could be appropriate in Uganda, and if so, inform the co-development of arts-in-health interventions.

Methods

Research design

We conducted an exploratory qualitative study using thematic analysis. Data were collected using semi-structured interviews with healthcare professionals and patients which focused on two main topics – i) the role of music and dance in Uganda; ii) the potential use of music and dance in CRD management in Uganda.

For peer review only

Setting

The study was conducted in the Makerere University Lung Institute (MLI) outpatient clinic in central Kampala, Uganda. This urban setting was selected due to the trusted relationships between the patients, clinical staff, and research teams, and the well-established academic relationship between the various research groups involved. Additionally, the institute has a well-established PR programme with informant groups that are knowledgeable regarding our topics of interest.

Participants

Purposeful convenience sampling was used to ensure a representative sample of relevant individuals and groups by gender, age, and religion, for both patients and healthcare professionals. Potential study participants were approached verbally (face-to-face or over the phone) after being identified by local staff and research team members working at the study location. Snowball sampling was used for further participant identification. Potential participants were provided with information (in appropriate language and format) about the study then given time to consider if they wanted to participate.

Inclusion criteria

- Adults aged ≥ 18 years with CRD who attend, previously attended, or have been invited to attend PR
- Health professionals who work with people with CRD
- Family members of adults with CRD

Exclusion criteria

- People unable to give informed consent
- People unable to participate due to physical or mental disabilities

Taster singing and dance sessions

Taster sessions took place, in the same week as interviews, to give participants an idea of how the sessions could be structured, and what experience participation. Trial singing sessions were delivered by Francis Mutesasira, a professional singing teacher. Francis is trained in the Singing for Lung Health methodology[18-20] and developed and ran the project 'Singing for Breathing (SFB) Uganda' for 3 months during 2018[21] at the MLI Kupumua House, which consisted of Singing for Lung Health techniques adapted to local songs and vocal exercises. Dance sessions were led by the lead physiotherapist for PR at the MLI who regularly integrates dance movements into his rehabilitation sessions, and KEJP who has developed and run dance sessions for people with long-term conditions. Taster sessions lasted between 20 and 40 minutes and took place in the MLI, in a large room normally used for the exercise component of PR sessions.

Data collection

Semi-structured interviews were conducted in October 2019, in the MLI, in private rooms, with no non-participants present. The topic guide was developed by reviewing conceptually related research projects conducted by the team and others (see 'Topic Guide' in supplement). Interviews focused on open-ended questions, with participant prompts to encourage further discussion on topics which appeared meaningful. Interviews were conducted by KP, LC and GN, in English or Luganda (predominant local language) depending on participant preference. If in Luganda, GN, an experienced qualitative researcher, translated simultaneously. Interviews were audio-recorded, and interviewers

For peer review only

documented immediate reflections following interviews. Interviewer participant relationships were established through relaxed introduction, and participants were informed the interviewers were health professionals, but not directly involved in the provision of their individual healthcare.

Structured observations of trial singing and dance sessions (see below) were conducted by KP, LC and GN (see structured observation proforma in supplement), and relevant documents analysed (see reference material listing in the supplementary materials), to support contextualisation and interpretation of interview data.

Daily meetings took place involving (depending on availability) GN, IK, KP, LC, RJ, BK, and WK, (DW from the UK) during which ongoing data collection and interpretation was discussed and triangulated with interview notes, structured observations and preparatory reference materials. This process aimed to facilitate understanding and inform the iterative development of ongoing data collection activities.

The participants were informed of the intention and focus of the research, and that their responses in no way influenced their ongoing care, rather that the intention was to inform the development of future interventions, if appropriate. Data were collected and handled as per COnsolidated criteria for REporting Qualitative studies (COREQ)-guidelines[22].

Data analysis

Interviews were transcribed verbatim. KP, LC and DW conducted a thematic analysis based on that described by Braun and Clarke[23] and Terry et al[24]. During phase 1, transcripts were read and re-read, with further listening and familiarising with interview recordings, interviewer reflections, and structured observations. Importantly, notes from discussions between GN, IK, KP, LC, RJ, BK, and WK made during data collection where used to facilitate understanding. Phase 2 included open free-coding, discussion, double-coding, cross-case analysis, and development of coding structure. As such the analysis was predominantly inductive in nature, though deductive elements were contributed by the semi-structured nature of using a topic guide. The coding structure was then refined into preliminary themes (phase 3), which were further discussed, refined, named, and agreed upon (phases 4 and 5). Participant validation was performed with staff members at the MLI. Given current COVID-19 restrictions, further patient participant validation was not performed, however, the clarity and inter-participant consistency of identified themes suggests that further participant validation would have been unlikely to dramatically alter findings. Theme saturation was achieved during the analysis, however given the exploratory nature of the study, all data were analysed. Coding and theme development used Microsoft Excel. Demographic and disease-specific information was sought from patient participants. Breathlessness scores were completed as an indication of disease-related functional impairment. This was selected given the heterogeneity of lung conditions represented, hence a generic rather than disease-specific assessment was appropriate. Additionally, breathlessness is a key assessment criterion for PR, hence relevant for the application of this study's findings.

Patient and Public involvement

Participant feedback collected during the 'Singing for Breathing Uganda' project evaluation, combined with consultation with patients attending respiratory clinics, prompted this study and informed the topic guide development. Additionally, the primary objective of the study is an exploration of patient and healthcare provider perspectives, hence patient and public involvement is at the core of this study.

For peer review only

Ethical approvals and consent

Ethical approval was granted by the Mulago Hospital Research and Ethics Committee (Reference number MHREC:1478) and the University of Plymouth Faculty Research Ethics and Integrity Committee (19/20-1164). All participants provided written informed consent. All research activities were conducted in accordance with the principles of the Declaration of Helsinki.

Results

Eleven patients and eight staff members were approached and recruited. Two further patients were approached and declined, stating they did not have time. Eight of the eleven participants were female, mean age 43 years (range 20-63). Regarding ethnicity, all participants were black Ugandan. All reported CRD, including PTBLD (x6), post 'infection' lung disease (x1), asthma (x2), COPD (x1), pulmonary fibrosis (x1). Modified Medical Research Council breathlessness scores (mMRC) ranged from 1 to 3 (mean 1.5). None of the patients used ambulatory oxygen. Various symptoms were reported, in keeping with their CRD, including breathlessness, cough, and physical activity limitations. All reported living in houses (rather than flats or 'other'). Two lived alone, nine were cohabiting with family. Seven were in paid employment, one was a student, and three were unemployed. Two patients were interviewed prior to taster sessions, while the other nine were interviewed after attending taster sessions, and two of these had also attended SFB Uganda the previous year. The eight healthcare professionals were four women, four men, mean age 41 years (range 29-59). Occupations represented were physiotherapist, respiratory researcher, administrator, carer (sister of a patient), nurse, and three doctors. Participant quotes below are preceded by a 'P' for patient, or 'S' for staff, and the participant number.

On most topics, perspectives between patients and healthcare professionals aligned closely. Our analysis identified four key themes: Music and dance as 1) central components of daily life; having an 2) Established role supporting health and wellbeing; and perceived as having 3) Strong therapeutic potential in respiratory conditions. However, the potential realisation of this 'strong potential' (theme 3)) was dependent upon theme 4) Modulating demographic considerations of cultural and religion, and age.

Theme 1: Music and dance as central components in daily life

Music and dance were described as omnipresent in the social, religious and cultural components of daily life in Uganda. Music and dance were largely inseparable from one another, and described as inclusive and participatory

> S1 *'music really is everywhere for us…Music is really part of our fabric as a society….when they play a song everyone identifies to and everyone is getting up and just dancing, it doesn't matter whether they're in a suit, they're jumping, dancing.'*
>
> and
>
> P4 *'my wife is a politician, when we go to rallies, they normally invite you to come and join them. We join them. Yes. We join them and dance.'*

Music's omnipresence was attributed to its multiple social functions, especially forging interpersonal connectivity

For peer review only

S1 *'dancing is a way of communing, of interacting with people. It is one of those things that bind people.'*, and P3 *'There is that kind of relationship, with people you sing with.'*

and

S3 *'music speaks to our situations or just that feeling of being together with people and you're singing and you're dancing.'*

A further function being information transfer

P11 *'Music is very important in our society because it gives messages, it educates through music you are able to know what is good, what is bad, what can be done, what happened in the past, what will happen in the future, all can be delivered through music'*

Participation in music and dance was generally referred to free-willed choice, however many also described a compulsion, as if driven by an external '*power of music*' that overcomes inhibiting factors

P9 '*[I] feel the music in [me] and [have] to dance*'.

Theme 2: Music and dance had an established role supporting health and wellbeing

Through their role in social, religious, and cultural aspects of life, music and dance were seen as already having established roles in supporting physical, mental, and social health. Such effects were often described as concurrent and interrelated.

Mental health

The most dominant established health-promoting roles related to mental health. Most patients identified this function

P3 *'[listening to music] you feel happy, you feel you are getting connected with the world that you are not seeing. If gives you some hopeful times. It gives a message. I keep with the message that gives some hope for the future.'*

And

P9 'instead of getting angry, [I] would try and find comfort in singing and dancing to control [my] anger'

Healthcare professionals also highlighted these functions, for patients, but also frequently described using music for stress relief and relaxation themselves. Psychological benefits were underpinned by enjoyment of participation

P8 'I feel nice when I'm singing'

and

S8 'When we are singing, of course you feel like you ... you feel that joy'

For peer review only

Physical health

Physical health improvements were mainly attributed to dance or exercise to music

> S4 '*now [dance] has been taken up as one of the things that's used for physical exercises*.'

A group of doctors had also started an afterwork exercise group where they use music for working out, with dance often seen as preferential to other forms of physical activity

> *S4 'I don't like walking, if I have a car, I will drive it. Even to the nearest distance. But I would do dancing as a physical activity and I would do it with love. Because I love it and I love music.'*

Compared with dance, purely physical health benefits were not frequently attributed to singing in its established (daily life) roles.

Social benefits

Social connectivity, as described in theme 1, supported social health and overlapped with mental health and wellbeing.

> P4 *'you are joining other people. You know, when you are a people orientated person, when you find people that are happy, you also become happy.'*

This was unpinned by the light-hearted enjoyable nature of music and dance participation

> S1 *'it's a fun activity. It's a fun bonding activity for us. Everyone dances whatever they have, silly strokes, and you're just laughing and having a good time.'*

Theme 3: Music and dance perceived as having strong therapeutic potential in respiratory disease management

Contextually appropriate

Perceived potential for successful integration was clear, largely due to the ubiquity (theme 1) and established roles (theme 2) of music and dance in promoting health and wellbeing

> S3 *'because of what our culture is we love partying, we love music, we love dancing, so I think if someone who is told that if you dance, if you sing it is going to improve your health I believe they will have no problem taking part of it.'*

Again, fundamental to the perceived potential, was enjoyment, and group participation

> P1 *'I think it's good to do it as a group. Because you encourage each other. I think it's also more fun, yeah, and then it makes it, you know, something which you've got faster, you move on longer.'*

Potential psychosocial impacts for patients' health conditions were highlighted

> S1 *'no amount of medicine can give you that human connection, which is a very important part of management.'*

For peer review only

Potential therapeutic mechanisms for physical improvements was also suggested by healthcare professionals

> S7 *'you go beyond your tidal volume, in terms of reaching out your respiratory effort … if they keep doing this song then every other time they have some incremental effort required of their respiratory muscles.'*

> and

> S8 *'I feel it helps because it requires breath control, breathing in, breathing out and at the end it is fun… And of course they are learning also how to sing, how to control their breath, which in their own way helps their healing process and of course coping with the environment.'*

The potential for delivery with minimal resource requirements was emphasised as an important factor, particularly where resources were most limited.

Health benefits

Potential health benefits for CRD patients related closely to the established roles of music and dance in wider society.

Physical benefits related to potential exercise training effects, which were seen as very important for people with CRD

> P3 *'with the singing, you feel the lungs, you know, get opened, you feel you breathe very well. You feel the body also, the body moves with the singing, and also dancing. It becomes more free.'*

Participants in the taster sessions reported improvements in symptoms

> P3 *'the sputum can come out very easily.'*

> And,

> S5 *'That their breathlessness has reduced so they can work a bit longer than they used to. Most of them, that's what they are saying.'*

Improvements in physical symptoms were intimately linked to psychological impacts

> P2 *'I was feeling a bit happier because I feel like I could breathe a bit better.'*

The role of social aspects within the taster sessions were noted as creating peer support,

> S6 *'It gives them courage and also helps them for the rehabilitation that they're supposed to do. Friends encourage each other to exercise. So it ends up being very, very efficient for them.'*

Enjoyment

Taster session participants were very positive about the experience, which was also noted by staff

> P6 *'People were excited, and they say that let us do this whenever we come. They have been so touched. At first we thought, what is this now? But at the end, it has been perfect.'*

For peer review only

As in theme 1, enjoyment was a facilitator of health impacts, and the novelty of the approach was noted positively. Additionally, as in theme 2, participants emphasised the need to adapt sessions to the specific participants of a session (see theme 4).

Already happening

The lead physiotherapist for pulmonary rehabilitation was already integrating dance into his sessions and reporting very positive responses

> S2 *'when you bring in a warm-up that is full of dancing and rhythmical, we see they are happy.'*

Also, one patient reported using music for disease specific self-management

> P4 *'when I get attacks, I go in my room, and what do I put on? The radio. So, what am I doing? Listening to music.'*

Theme 4: Modulating demographic considerations

Participants emphasised that, for successful implementation, activities or interventions would need to be adapted to the specific participants of any one group, and the group itself. Key factors for consideration to ensure appropriate content included culture and religion, age, gender, and extent of urbanisation. These factors were important for two reasons. Firstly, to ensure that no member of the group felt uncomfortable or excluded. Secondly, responsive contextualisation was seen as a tool to optimise engagement and enjoyment – by selecting songs, music or dances that had cultural or historical significance for the group, a sense of collective identity could be established. This would facilitate interpersonal interactions based on shared experience and knowledge. Such an approach was almost presented as being obvious by participants, as this was how music and dance are used in Uganda more broadly. Contextualisation and personalisation were seen as being part of the essence of music and dance themselves

> S2 *'dancing has no formula, it has no pattern. It's not a matter of, oh you must conform. Each one has their own dance. I believe that if I was dancing with you, you have your own style of dancing, and I have my own style of dancing.'*

Culture and religion

For the study respondents, the concepts of culture and religion were interrelated. The terms 'culture' or 'traditional' were often used in reference to traditional tribal practices, beliefs and identities, while 'religion' referred to world religions (Christian, Muslim, or atheist/agnostic)

> S2 '*those folk songs, traditional, that people can engage to traditional dances that train from tribe to tribe.'*

Culture is extremely important in Uganda, and music and dance are core to these aspects of daily life (Theme 1)

> P1 '*There is no culture in Uganda where there isn't dancing.'*

However, expressions and norms differ

For peer review only

S1 *'every culture, every part of this country has a different kind of dance.'*

Similarly, religion is very important. In Kampala the majority of people identify as Christian, of various denominations, with a smaller but significant proportion follow Islam (14%)[25]. Music and dance are prominent in religious practices and contexts

S8 *'We rarely go direct into praising, praying without singing, without dancing… of course giving glory to god, giving your leg, you are giving your arms, so why not dance.'*

and

P6 *'for the Christians, they are used to singing, because in churches, Protestants do sing. Catholics do sing. Adventists do sing. Born again, most of the people... even the Witch crafts they have their praise, they praise. Yeah. People are used to singing. And Muslims sometimes they do sing.'*

It was suggested that Muslim participants might find singing and dance less acceptable, however, the one Muslim participant was positive about the taster session

P9 *'the dancing helps [me] so much, it's so uplifting.'*

Cultural norms were also highlighted such as issues around exposing parts of the body in close proximity, or how social status may influence acceptability and participation

S7 *'"I'm a Sheikh. I'm a Bishop. I'm a very tough father at home." You know, that kind of person who has a very cut-out social role they probably won't come to sing so much… Such a patient might think that singing might be lowering their social role.'*

Differences between urban and rural norms were highlighted

P1 *'in the rural areas dancing is more associated to ceremony party, not a day to day.'* P1 *rural areas are more conservative 'dresses that are longer, skirts like longer, no slits.'*

Age

As per Theme 1, music and dance were described as having multiple functions, the predominant function for an individual was seen to be modulated by age

S7 *'the old people they still love their music. Where it's a story telling song or it's something to harmonise and move or to advance excitement at a party. Yeah generally the young people of course they love it. Dancing and shaking around.'*

However, age was not seen as a barrier

S7 *'[older people] like dancing, and quite many of the old they get excited and dance.'*

The perception of others

The importance of these demographic factors also related to how participation might be seen by others, including family, friends and the wider community. Overall, if the activities were clearly being delivered in a therapeutic capacity, participants felt that social acceptance would be high

P8 *'[my family members] are excited, they want the results afterwards.'*

For peer review only

Improving acceptability

Given these considerations, participants suggested various ways to optimise acceptability. An emphasis on dance being physical exercise was proposed. Additionally, clearly stating the intended therapeutic benefits was important. Similarly, the therapeutic intention of the singing was important, and this was well communicated during the taster sessions

> P8 *'The singing, it is a different kind of singing also, yes, not all songs. But just get songs that push the lungs, expands the lungs, makes the lungs okay, yeah. And the dancing, it depends on the strokes you make, there are dance strokes that stretches the muscles.'*

Health professionals felt acceptance would be more forthcoming if a clear evidence base was also provided. And using the local languages was described by one participant as a method of increasing engagement through cultural identification

> S7 *'they will be more interested in the songs which are done in the local languages. They are richer in terms of connection with the audience.'*

Appropriate song selection would be facilitated by using secular music and co-creating session content specific to the group. This approach worked well in the taster sessions

> P6 *'we sang our national anthem of Uganda. It is for all of us.'*

Of note, although the demographic variables highlighted were considered important by study participants, they were eclectic in their music preferences, with culture and religious norms seen as informing, rather than limiting

> S2 *'The trend is from cultural, traditional, to any pattern somebody wishes to.'*

Additional implementation factors

In addition to demographic considerations, there was a broad appreciation that session content would also be adapted to the physical capacity of individual participants. Participants in the taster sessions felt such adaption took place successfully

> P1 *'for those who are a bit weak, to know that they can rest, when the body feels that it is tired. I thought that that was good.'*

Also, financial and time costs would need consideration to facilitate attendance. Suggestions included having sessions a maximum of once weekly, and subsidising travel costs, to ensure sessions led to net benefit rather than risk contributing to already strained financial situations.

Discussion

The results of this in-depth qualitative study show that music and dance are core components of daily life in Uganda. Study participants felt that participation supports both collective and individual health and wellbeing. These functions supported the perspective, from patients and healthcare professionals, that music and dance had great potential to improve elements of physical, mental and social health and wellbeing, for people with CRD. Individuals who had prior experience of arts-in-health activities, or who participated in taster sessions, were very enthusiastic about the concept.

For peer review only

They highlighted important factors for consideration for co-development and successful implementation primarily related to culture and religion, and age.

This study has multiple strengths. Firstly, to our knowledge it is the first to explore this topic. Secondly, the wide-ranging expertise of the research team strengthened interpretation. Thirdly, using in-depth interviews, triangulated with structured observations and key documentation, enabled a detailed, highly contextualised exploration of themes. Fourthly, purposeful convenience sampling ensured appropriate representation from relevant stakeholders.

Certain study limitations are important to discuss. Firstly, being a single site study, the transferability of findings cannot be ascertained, particularly regarding areas of Uganda outside of Kampala, where social and cultural groups and norms are likely to differ. However, Kampala is a district that has a mixture of all tribes in Uganda, and the MLI is a specialist centre, receiving referrals from all over the country. Secondly, COVID-19 pandemic restrictions relating to certain group activities, including singing and exercise[26] are currently in place, and these data were collected prior to the pandemic, hence, the COVID-19 related concerns may change the experience of group activities such as music and dance when they are considered safe to recommence. Additionally, here we report participants' perceptions regarding potential health benefits, and although in general, beneficial effects of similar interventions have been demonstrated, formal research and evaluation of this specific intervention is still required.

Although no other studies have investigated this topic in low-resource settings, our findings echo those of research in related contexts. Research on Singing for Lung Health (SLH) in the UK suggests participants perceive a range of physical, psychological and social benefits in keeping with our findings[11 18 20 27-29]. Similarly, studies regarding the perceived impacts of dance for people with long-term respiratory conditions in the UK and Canada identify a range of biopsychosocial benefits[10 12 30]. Additionally, an evaluation of SFB Uganda, a singing project for people with CRD in Uganda, provided anecdotal reports that participation was enjoyed[21], and our findings are broadly in keeping with the evaluation of SFB. Similarly, anecdotal experience of related singing and dance projects for people with long-term respiratory conditions in other low-resource settings have been enjoyed with participants reporting a range of biopsychosocial benefits[31]. A study of culturally adapted PR in the MLI also showed high-levels of acceptability[8]. Importantly, in each situation described, contextual adaptation and co-development of activities appears crucial to success. Interestingly, there was close alignment regarding responses from patients and healthcare professionals. This may be expected regarding the general role of music and dance in Uganda but was also the case in relation to potential therapeutic interventions. The main differences between the groups were healthcare professionals discussing potential therapeutic mechanisms in more depth and emphasising the requirement for an evidence base to increase acceptance.

The current COVID-19 pandemic has necessitated certain restrictions on group activities such as singing[26] and dance, which are likely to impact the potential application of these findings in the short-term. However, developments including widespread immunisations, infection control measures, and remotely delivered singing and dance interventions[13 32] may help reduce risk. Additionally, although the majority of participants were highly positive about participation, there were exceptions. As such, music and dance could be used as optional adjuncts to optimise uptake and completion of established, evidence-based respiratory management approaches such as PR.

For peer review only

Conclusions

Long-term respiratory conditions are common in Uganda, causing a high burden of morbidity and mortality. Low-cost, low-resource, interventions are of wide-reaching interest. Our findings suggest people with CRD, and healthcare professionals, see a great deal of potential for the use of music and dance as adjunctive roles to PR, or possibly be delivered as independent activities within CRD management. Building on established therapeutic roles of music and dance in wider Ugandan society, through co-produced intervention development specific to respiratory patients, appears to be a viable route for intervention development. These findings are important for developing arts-in-health interventions in Uganda and beyond.

Information on Researchers:

First Author: KEJP is a 35-year-old male respiratory physician who previously worked as a dancer and dance teacher, with experience leading community dance and dance for people with respiratory disease in the UK, South America, South Africa, Uganda and the Kyrgyz Republic. He lived in Uganda for 8 months during 2015 and 2016, and South Africa for 2 years. KEJP has received training in qualitative research methods from the Imperial College, University College London, and through self-directed learning. He is currently completing a PhD at Imperial College, using qualitative and quantitative methods. Qualifications: MBChB, BSc, MRCP, EADTMH, DPMSA.

DW has extensive experience of qualitative research methods and applied health services research in the UK and international settings and co-designed the research methods for this study. Qualifications MA

Mark William Orme, 30yo, male, senior researcher and research manager for global health project on pulmonary rehabilitation in LMIC including Uganda, PhD. Experience and informal training in qualitative design (interviews and focus groups), conduct (interviews) and analysis (thematic).

Evelyn Brakema, female, 31 years old from the Netherlands, PhD-candidate Global Health (implementation of interventions targeting chronic respiratory disease in Uganda and other low-resource settings) & MD in training for family physician (please decide if relevant). Experience and formal training in qualitative research, particularly from the Horizon 2020 FRESH AIR Project.

Co-authors: LC, DW, GN, IK, BK, EB, MO, DF, NS all have training and experience in qualitative research methods.

Senior author: WK has extensive qualitative and quantitative research experience.

References

1. Ferkol T, Schraufnagel D. The global burden of respiratory disease. *Annals of the American Thoracic Society* 2014;11(3):404-6. doi: 10.1513/AnnalsATS.201311-405PS
2. van Zyl Smit RN, Pai M, Yew WW, et al. Global lung health: the colliding epidemics of tuberculosis, tobacco smoking, HIV and COPD. *The European respiratory journal* 2010;35(1):27-33. doi: 10.1183/09031936.00072909 [published Online First: 2010/01/02]

For peer review only

3. World Health Organisation. GLOBAL STATUS REPORT on noncommunicable diseases 201. Switzerland The WHO, 2014.
4. Salvi S. The silent epidemic of COPD in Africa. *The Lancet Global health* 2015;3(1):e6-7. doi: 10.1016/S2214-109X(14)70359-6 [published Online First: 2014/12/30]
5. van Gemert F, Kirenga B, Chavannes N, et al. Prevalence of chronic obstructive pulmonary disease and associated risk factors in Uganda (FRESH AIR Uganda): a prospective cross-sectional observational study. *The Lancet Global health* 2015;3(1):e44-51. doi: 10.1016/S2214-109X(14)70337-7
6. van Gemert F, van der Molen T, Jones R, et al. The impact of asthma and COPD in sub-Saharan Africa. *Prim Care Respir J* 2011;20(3):240-8. doi: 10.4104/pcrj.2011.00027 [published Online First: 2011/04/22]
7. Jones R, Muyinda H, Nyakoojo G, et al. Does pulmonary rehabilitation alter patients' experiences of living with chronic respiratory disease? A qualitative study. *Int J Chron Obstruct Pulmon Dis* 2018;13:2375-85. doi: 10.2147/COPD.S165623 [published Online First: 2018/08/21]
8. Jones R, Kirenga BJ, Katagira W, et al. A pre-post intervention study of pulmonary rehabilitation for adults with post-tuberculosis lung disease in Uganda. *Int J Chron Obstruct Pulmon Dis* 2017;12:3533-39. doi: 10.2147/COPD.S146659 [published Online First: 2017/12/23]
9. Philip K, Lewis A, Hopkinson NS. Music and dance in chronic lung disease. *Breathe* 2019;15(2):116-20. doi: 10.1183/20734735.0007-2019
10. Wshah A, Butler S, Patterson K, et al. "Let's Boogie": FEASIBILITY OF A DANCE INTERVENTION IN PATIENTS WITH CHRONIC OBSTRUCTIVE PULMONARY DISEASE. *J Cardiopulm Rehabil Prev* 2019;39(5):E14-E19. doi: 10.1097/HCR.0000000000000428 [published Online First: 2019/08/30]
11. Kaasgaard M, Rasmussen DB, Ottesen AL, et al. Sing-a-Lung: Group singing as training modality in pulmonary rehabilitation for patients with Chronic Obstructive Pulmonary Disease (COPD): A multicenter, cluster-randomised, non-inferiority controlled trial. *ERJ* 2020;56 doi: 10.1183/13993003.congress-2020.4663
12. Philip KEJ, Lewis A, Williams S, et al. Dance for people with chronic respiratory disease: a qualitative study. *BMJ open* 2020;10(10):e038719. doi: 10.1136/bmjopen-2020-038719 [published Online First: 2020/10/15]
13. Philip KEJ, Lewis A, Jeffery E, et al. Moving Singing for Lung Health online: experience from a randomised controlled trial. *medRxiv* 2020
14. Philip KEJ, Lewis A, Buttery SC, et al. The physiological demands of Singing for Lung Health compared to treadmill walking. *medRxiv* 2020 doi: 10.1101/2020.12.08.20245746
15. Lee AL, Desveaux L, Goldstein RS, et al. Distractive Auditory Stimuli in the Form of Music in Individuals With COPD: A Systematic Review. *Chest* 2015;148(2):417-29. doi: 10.1378/chest.14-2168 [published Online First: 2015/03/06]
16. Fancourt DaF, S.,. What is the evidence on the role of the arts in improving health and well-being? A scoping review (2019): World Health Organisation, 2019.
17. Daykin N, Gray K, McCree M, et al. Creative and credible evaluation for arts, health and well-being: opportunities and challenges of co-production. *Arts & Health* 2017;9:123-38. doi: 10.1080/17533015.2016.1206948
18. Lewis A, Cave P, Hopkinson NS. Singing for Lung Health: service evaluation of the British Lung Foundation programme. *Perspect Public Health* 2018;138(4):215-22. doi: 10.1177/1757913918774079 [published Online First: 2018/05/15]
19. Lewis A, Cave P, Hopkinson NS. Singing for Lung Health: a qualitative assessment of a British Lung Foundation programme for group leaders. *BMJ open respiratory research* 2017;4(1):e000216. doi: 10.1136/bmjresp-2017-000216
20. Lewis A, Cave P, Stern M, et al. Singing for Lung Health-a systematic review of the literature and consensus statement. *NPJ primary care respiratory medicine* 2016;26:16080. doi: 10.1038/npjpcrm.2016.80

For peer review only

21. Downes C, Philip KEJ, Lewis A, et al. Singing for Breathing Uganda: Group singing for people with chronic lung disease in Kampala. *Journal of Applied Arts & Health* 2019;10(2):219-28. doi: 10.1386/jaah.10.2.219_1
22. Tong A, Sainsbury P, Craig J. Consolidated criteria for reporting qualitative research (COREQ): a 32-item checklist for interviews and focus groups. *Int J Qual Health Care* 2007;19(6):349-57. doi: 10.1093/intqhc/mzm042 [published Online First: 2007/09/18]
23. Clarke V, Braun, V., Hayfield, N.,. Thematic Analysis. In: Smith JA., ed. Qualitative Psychology: SAGE 2015:222-48.
24. Terry G, Hayfield, N., Clarke, V., Braun, V.,. Thematic Analysis in: The SAGE Handbook of Qualitative Research in Psychology. London: SAGE Publications Ltd 2017:17-36.
25. Uganda Bureau of Statistics. 2014 Census, 2018.
26. Philip KEJ, Lewis A, Buttery SC, et al. Aerosol Transmission of SARS-CoV-2: Inhalation as Well as Exhalation Matters for COVID-19. *American journal of respiratory and critical care medicine* 2021 doi: 10.1164/rccm.202012-4445LE [published Online First: 2021/02/03]
27. Philip KE, Lewis A, Jeffery E, et al. Moving singing for lung health online in response to COVID-19: experience from a randomised controlled trial. *BMJ open respiratory research* 2020;7(1) doi: 10.1136/bmjresp-2020-000737 [published Online First: 2020/11/27]
28. Lord VM, Hume VJ, Kelly JL, et al. Singing classes for chronic obstructive pulmonary disease: a randomized controlled trial. *BMC pulmonary medicine* 2012;12:69. doi: 10.1186/1471-2466-12-69
29. Lord VM, Cave P, Hume VJ, et al. Singing teaching as a therapy for chronic respiratory disease--a randomised controlled trial and qualitative evaluation. *BMC pulmonary medicine* 2010;10:41. doi: 10.1186/1471-2466-10-41
30. Harrison SL BK, Edwards J, McFaull V, McLusky S, Russell A, Williams G, Williams S. DANCE FOR PEOPLE WITH CHRONIC BREATHLESSNESS: A FEASIBILITY STUDY. Thorax, 2019.
31. Philip KEJ, Katagira W, Jones R. Dance for Respiratory Patients in Low-Resource Settings. *Jama* 2020;324(10):921-22. doi: 10.1001/jama.2020.15426 [published Online First: 2020/09/09]
32. Philip K, Lewis, A., Harrison, S.,. Singing and Dance for People with Chronic Breathlessness during the COVID-19 pandemic: American Thoracic Society 2020 [Available from: https://www.thoracic.org/members/assemblies/assemblies/pr/quarterly-bite/singing-and-dance-for-people-with-chronic-breathlessness-during-the-covid-19-pandemic.php accessed 29/07/2020 2020.

For peer review only

Topic guide interviews –family members

1. How much do you listen to music at home/work?
    a. What type of music?
    b. Who do you listen with?
    c. How do you listen to music (probe for devices/access)
    d. How often?
    e. Do you sing along?
    f. How does music make you feel?
2. Tell us about dancing:
    a. How much do you dance/(other term?) – how often?
    b. What type of dancing?
    c. Who with?
    d. How does it feel to dance?
    e. Does your condition affect your dancing in any way?
    f. What do your friends/family think about that?
3. How is music and dance regarded in your family?
    a. And in your community?
    b. Do people think it is important? Why?
4. What do you think about singing and dancing as a way to improve health for people like your family member?
5. What do you think other people in your community/family would think about that?
6. Would their opinion make any difference to whether your family member might take part?

IF RESPONSES ARE POSTIIVE TO QS 4-6 THEN ASK

7. What do you think could be any difficulties for your family member in doing singing or dance to improve health?
8. What would be the things that might help them?

For peer review only - http://bmjopen.bmj.com/site/about/guidelines.xhtml

Kupumua Structured Observation

Sheet 1

Trial session:

Date:

Observer:

Location:

People present:

Observation of an active session (could be singing, dancing, PR or PR plus music/dance)

Observation	Session type:
1. Body language	
2. Facial expressions	
3. Speech/expression	
4. Interactions between peers	
5. Interactions with staff	
6. Physical involvement with music, singing, dancing	
7. Disease related behaviour (short of breath, coughing, fatigue, resting periods,	
8. Role within the group. Passive/active. Lead/follow.	
9. Reflexive researcher responses	

For peer review only - http://bmjopen.bmj.com/site/about/guidelines.xhtml

For peer review only

For peer review only - http://bmjopen.bmj.com/site/about/guidelines.xhtml

COREQ (COnsolidated criteria for REporting Qualitative research) Checklist

A checklist of items that should be included in reports of qualitative research. You must report the page number in your manuscript where you consider each of the items listed in this checklist. If you have not included this information, either revise your manuscript accordingly before submitting or note N/A.

Topic	Item No.	Guide Questions/Description	Reported on Page No.
**Domain 1: Research team and reflexivity**			
Personal characteristics			
Interviewer/facilitator	1	Which author/s conducted the interview or focus group?	
Credentials	2	What were the researcher's credentials? E.g. PhD, MD	
Occupation	3	What was their occupation at the time of the study?	
Gender	4	Was the researcher male or female?	
Experience and training	5	What experience or training did the researcher have?	
Relationship with participants			
Relationship established	6	Was a relationship established prior to study commencement?	
Participant knowledge of the interviewer	7	What did the participants know about the researcher? e.g. personal goals, reasons for doing the research	
Interviewer characteristics	8	What characteristics were reported about the inter viewer/facilitator? e.g. Bias, assumptions, reasons and interests in the research topic	
**Domain 2: Study design**			
Theoretical framework			
Methodological orientation and Theory	9	What methodological orientation was stated to underpin the study? e.g. grounded theory, discourse analysis, ethnography, phenomenology, content analysis	
Participant selection			
Sampling	10	How were participants selected? e.g. purposive, convenience, consecutive, snowball	
Method of approach	11	How were participants approached? e.g. face-to-face, telephone, mail, email	
Sample size	12	How many participants were in the study?	
Non-participation	13	How many people refused to participate or dropped out? Reasons?	
Setting			
Setting of data collection	14	Where was the data collected? e.g. home, clinic, workplace	
Presence of non-participants	15	Was anyone else present besides the participants and researchers?	
Description of sample	16	What are the important characteristics of the sample? e.g. demographic data, date	
Data collection			
Interview guide	17	Were questions, prompts, guides provided by the authors? Was it pilot tested?	
Repeat interviews	18	Were repeat inter views carried out? If yes, how many?	
Audio/visual recording	19	Did the research use audio or visual recording to collect the data?	
Field notes	20	Were field notes made during and/or after the inter view or focus group?	
Duration	21	What was the duration of the inter views or focus group?	
Data saturation	22	Was data saturation discussed?	
Transcripts returned	23	Were transcripts returned to participants for comment and/or	

For peer review only

Topic	Item No.	Guide Questions/Description	Reported on Page No.
		correction?	
**Domain 3: analysis and findings**			
Data analysis			
Number of data coders	24	How many data coders coded the data?	
Description of the coding tree	25	Did authors provide a description of the coding tree?	
Derivation of themes	26	Were themes identified in advance or derived from the data?	
Software	27	What software, if applicable, was used to manage the data?	
Participant checking	28	Did participants provide feedback on the findings?	
Reporting			
Quotations presented	29	Were participant quotations presented to illustrate the themes/findings? Was each quotation identified? e.g. participant number	
Data and findings consistent	30	Was there consistency between the data presented and the findings?	
Clarity of major themes	31	Were major themes clearly presented in the findings?	
Clarity of minor themes	32	Is there a description of diverse cases or discussion of minor themes?	

Developed from: Tong A, Sainsbury P, Craig J. Consolidated criteria for reporting qualitative research (COREQ): a 32-item checklist for interviews and focus groups. *International Journal for Quality in Health Care*. 2007. Volume 19, Number 6: pp. 349 – 357

Once you have completed this checklist, please save a copy and upload it as part of your submission. DO NOT include this checklist as part of the main manuscript document. It must be uploaded as a separate file.

For peer review only

BMJ Open

Music and Dance in respiratory disease management in Uganda: A qualitative study of patient and healthcare professional perspectives

Journal:	*BMJ Open*
Manuscript ID	bmjopen-2021-053189.R1
Article Type:	Original research
Date Submitted by the Author:	14-Aug-2021

Complete List of Authors:	Philip, Keir; Imperial College London; NIHR Imperial Biomedical Research Centre<br>Cartwright, Lucy L; University of Plymouth<br>Westlake, Debra; University of Plymouth<br>Nyakoojo, Grace; Makerere University Lung Institute<br>Kimuli, Ivan; Makerere University Lung Institute<br>Kirenga, Bruce; Makerere University Lung Institute<br>Brakema, Evelyn A; Leiden University Medical Center, Department of Public Health and Primary Care<br>Orme, Mark; Department of Respiratory Sciences, University of Leicester (United Kingdom); Centre for Exercise and Rehabilitation Science, NIHR Leicester Biomedical Research Centre – Respiratory, University Hospitals of Leicester NHS Trust - Leicester (United Kingdom)<br>Fancourt, Daisy; University College London, Department of Behavioural Science and Health<br>Hopkinson, Nicholas; Imperial College London<br>Jones, Rupert; University of Plymouth, PUPSMD<br>Katagira, Winceslaus; Makerere University Lung Institute

**Primary Subject Heading**:	Respiratory medicine
Secondary Subject Heading:	Global health, Patient-centred medicine, Qualitative research, Rehabilitation medicine, Sociology
Keywords:	Respiratory infections < THORACIC MEDICINE, Tuberculosis < INFECTIOUS DISEASES, Thoracic medicine < INTERNAL MEDICINE, Rehabilitation medicine < INTERNAL MEDICINE, Chronic airways disease < THORACIC MEDICINE

SCHOLARONE™
Manuscripts

I, the Submitting Author has the right to grant and does grant on behalf of all authors of the Work (as defined in the below author licence), an exclusive licence and/or a non-exclusive licence for contributions from authors who are: i) UK Crown employees; ii) where BMJ has agreed a CC-BY licence shall apply, and/or iii) in accordance with the terms applicable for US Federal Government officers or employees acting as part of their official duties; on a worldwide, perpetual, irrevocable, royalty-free basis to BMJ Publishing Group Ltd ("BMJ") its licensees and where the relevant Journal is co-owned by BMJ to the co-owners of the Journal, to publish the Work in this journal and any other BMJ products and to exploit all rights, as set out in our licence.

The Submitting Author accepts and understands that any supply made under these terms is made by BMJ to the Submitting Author unless you are acting as an employee on behalf of your employer or a postgraduate student of an affiliated institution which is paying any applicable article publishing charge ("APC") for Open Access articles. Where the Submitting Author wishes to make the Work available on an Open Access basis (and intends to pay the relevant APC), the terms of reuse of such Open Access shall be governed by a Creative Commons licence – details of these licences and which Creative Commons licence will apply to this Work are set out in our licence referred to above.

Other than as permitted in any relevant BMJ Author's Self Archiving Policies, I confirm this Work has not been accepted for publication elsewhere, is not being considered for publication elsewhere and does not duplicate material already published. I confirm all authors consent to publication of this Work and authorise the granting of this licence.

For peer review only

Music and Dance in respiratory disease management in Uganda: A qualitative study of patient and healthcare professional perspectives

For peer review only

Authors: Keir EJ Philip[1,2*], Lucy L Cartwright[3], Debra Westlake[3], Grace Nyakoojo[4], Ivan Kimuli[4], Bruce Kirenga[4], Evelyn A Brakema[5], Mark W Orme[6], Daisy Fancourt[7], Nicholas S Hopkinson[1], Rupert Jones[8,4], Winceslaus Katagira[4]

Affiliations

1) National Heart and Lung Institute, Imperial College London, London, United Kingdom
2) NIHR Imperial Biomedical Research Centre, London, United Kingdom
3) Faculty of Health, University of Plymouth, Plymouth, United Kingdom
4) Makerere University Lung Institute, Kampala, Uganda
5) Department of Public Health and Primary Care, Leiden University Medical Center, Leiden, the Netherlands
6) Department of Respiratory Sciences, University of Leicester, United Kingdom
7) Department of Behavioural Science and Health, University College London, United Kingdom
8) Research and Knowledge Exchange, Plymouth Marjon University, United Kingdom

*Corresponding author:

Dr Keir Philip

Email: k.philip@imperial.ac.uk

Physical address: The Respiratory Muscle Laboratory, Royal Brompton Hospital, Fulham Road, London SW3 6HP, United Kingdom

Main Manuscript word Count: 5147

Funding:

KP was supported by National Institute for Health Research Academic Clinical Fellowship award and the Imperial College Clinician Investigator Scholarship. The funders had no say in the design and conduct of the study; collection, management, analysis, and interpretation of the data; preparation, review, or approval of the manuscript; and decision to submit the manuscript for publication. This publication presents independent research. The views expressed are those of the authors and not necessarily those of the NHS, the NIHR or the Department of Health and Social Care. DF was supported by the Wellcome Trust [205407/Z/16/Z].

The research leading to these results has received support from the EU Research and Innovation program Horizon2020 (H2020 European Research Council) under grant agreement no. 680997, part of the Health, Medical Research and the Challenge of Ageing Horizon 2020 action, and funding from the University of Plymouth Global Challenges Research Fund (GCRF) internal fund.

Acknowledgements

We would like to thank the study participants for their time and effort, Stuart Blatston at the 625 for supporting logistical requirements of the project; Francis Mutesasira for delivering the singing sessions; and Richard Kasiita for his contributions.

For peer review only

Competing interests:

Rupert Jones declares grants unrelated to this study from Astra Zeneca, Glaxo Smith Kline and Novartis and personal fees for consultancy, speakers fees or travel support from Astra Zeneca, Boehringer Ingelheim, Glaxo Smith Kline, Novartis, Nutricia and OPRI

No other authors declare conflicts.

Data availability

All data relevant to the study are included in the article or uploaded as supplementary information. No additional data available.

Contributorship statement:

All authors meet the criteria for authorship as recommended by the International Committee of Medical Journal Editors. KEJP, RJ, WK, and BK had the original idea for the study. All authors (KEJP, LLC, DW, GN, IK, BK, EAB, MWO, DF, NSH, RJ, WK) were involved in designing the study. DW provided guidance on qualitative methods including design and analysis. KEJP, LLC, GN conducted the interviews. KEJP, LLC, DW, GN, RJ and WK conducted the analysis, which was then discussed with the other authors and refined. The first draft of the manuscript was written by KEJP. All authors (KEJP, LLC, DW, GN, IK, BK, EAB, MWO, DF, NSH, RJ, WK) read, contributed to, and agreed on the final manuscript draft.

Abstract: (246 words)

Introduction:

Music and dance are increasingly used as adjunctive arts-in-health interventions in high-income settings, with a growing body of research suggesting biopsychosocial benefits. Such low-cost, low-resource interventions may have application in low-resource settings such as Uganda. However, research on perceptions of patients and healthcare professionals regarding such approaches is lacking.

Methods

We delivered sample sessions of music and dance for chronic respiratory disease (CRD) to patients and healthcare professionals. Seven participants took part in one singing and dance sample session. One patient completed only the dance session. We then conducted an exploratory qualitative study, using thematic analysis of semi-structured interviews with the healthcare professionals and patients regarding i) the role of music and dance in Ugandan life and ii) the perceived acceptability and feasibility of using music and dance in CRD management in Uganda.

Results

We interviewed 19 participants, made up of eleven patients with long-term respiratory conditions and eight healthcare professionals, who were selected by purposeful convenience sampling. Four key themes were identified from interview analysis: Music and dance: 1) were central components of daily life; 2) had an established role supporting health and wellbeing; 3) had strong therapeutic potential in respiratory disease management. A fourth theme was: 4) the importance of modulating demographic considerations of culture, religion and age.

Conclusion

Music and dance are central to life in Uganda, with established roles supporting health and wellbeing. These roles could be built on in the development of music and dance interventions as adjuncts to established components of CRD disease management like pulmonary rehabilitation. Through consideration of key contextual factors, and co-development and adaptation of interventions, such approaches are likely to be well received.

Strengths and limitations of this study

- This is the first study to explore patient and healthcare perspectives regarding the use of music and dance in respiratory disease management in Uganda.
- Using in-depth interviews, triangulated with structured observations and key documentation, enabled a detailed, highly contextualised exploration of themes.
- Purposeful convenience sampling ensured appropriate representation from relevant stakeholders.
- As a single site study, the transferability of findings cannot be ascertained

For peer review only

- The COVID-19 pandemic, and related restrictions regarding group activities including singing and exercise, may have influenced perspectives on participating in singing and dance activities.

For peer review only

Introduction

Chronic Respiratory Diseases (CRD) such as post-tuberculosis lung disease (PTBLD), asthma, and chronic obstructive pulmonary disease (COPD) are leading causes of morbidity and mortality globally[1]. The burden of respiratory disease disproportionately affects people in low and middle-income countries, where over 90% of global respiratory deaths occur[1-3], and is predominantly caused by smoking, respiratory infections, biomass smoke exposure, poor nutrition and air pollution[2]. Prevalence data are limited from Africa[4], however in Uganda specifically, research suggests that CRD are common[5 6]. People with CRD in Africa suffer from a high burden of symptoms amplified by social isolation, economic disadvantage and stigmatisation[7] related to their symptoms. Physical exercise training and self-management education are important components of CRD management, with the interaction between symptoms, inactivity, and psychological impairment key factors in the 'cycle of decline' see Jones et al (2018)[7]. There is interest in developing locally adapted, low-cost high-impact interventions in this patient group, for example a recent programme development study has shown that pulmonary rehabilitation (PR) is feasible and improves quality of life and exercise capacity in people with PTBLD in Uganda[8].

Singing and dance have become increasingly popular adjuncts to conventional disease management strategies for people with long-term respiratory conditions in the UK[9] and other high-income countries[10]. Existing research suggests participants experience a range of biopsychosocial benefits including those related to physical performance, mental health and wellbeing, and social isolation[9 11-14]. Music as distractive auditory stimuli during exercise training for people with CRD can reduce breathlessness and increase exercise capacity[15]. Although a large and growing body of research supports using the arts to support health and wellbeing[16], research in low resource settings is limited. Additionally, co-production of arts-in-health activities is widely appreciated as central to the successful development and adaptation of interventions[17]. Furthermore, a recent systematic review and meta-analysis of critical factors required for successful implementation of lung health interventions in low and middle-income countries emphasised the importance of ensuring compatibility with the local context and understanding needs of local users[18]. Through the engagement of key stakeholders, including staff, patients and family members, such activities have the potential to utilise pre-existing sociocultural resources and minimise dependence on additional external funding or resources.

Data from this study examined the perspective of people with long-term respiratory conditions and respiratory healthcare professionals in Uganda to answer the following questions:

- What are the current roles of music and dance in general life?
- Would the use of music and/or dance in the management of long-term respiratory conditions be acceptable and feasible?

Answering these questions is important to establish if such approaches could be appropriate in Uganda, and if so, inform the co-development of arts-in-health interventions.

Methods

Research design

We conducted an exploratory qualitative study using thematic analysis. Data were collected using semi-structured interviews with healthcare professionals and patients which focused on two main

For peer review only - http://bmjopen.bmj.com/site/about/guidelines.xhtml

topics – i) the role of music and dance in Uganda; ii) the potential use of music and dance in CRD management in Uganda.

Setting

The study was conducted in the Makerere University Lung Institute (MLI) outpatient clinic in central Kampala, Uganda. This urban setting was selected due to the trusted relationships between the patients, clinical staff, and research teams, and the well-established academic relationship between the various research groups involved. Additionally, the institute has a well-established PR programme with informant groups that are knowledgeable regarding our topics of interest.

Participants

Purposeful convenience sampling was used to ensure a representative sample of relevant individuals and groups by gender, age, and religion, for both patients and healthcare professionals. Potential study participants were approached verbally (face-to-face or over the phone) after being identified by local staff and research team members working at the study location. Snowball sampling was used for further participant identification. Potential participants were provided with information (in appropriate language and format) about the study then given time to consider if they wanted to participate.

Inclusion criteria

- Adults aged ≥ 18 years with CRD who attend, previously attended, or have been invited to attend PR
- Health professionals who work with people with CRD
- Family members of adults with CRD

Exclusion criteria

- People unable to give informed consent
- People unable to participate due to physical or mental disabilities

Sample singing and dance sessions

Sample sessions took place, in the same week as interviews, to give participants an idea of how the sessions could be structured, and what experience participation. Trial singing sessions were delivered by Francis Mutesasira, a professional singing teacher. Francis is trained in the Singing for Lung Health methodology[19-21] and developed and ran the project 'Singing for Breathing (SFB) Uganda' for 3 months during 2018[22] at the MLI Kupumua House, which consisted of Singing for Lung Health (Singing for lung health | British Lung Foundation (blf.org.uk)) techniques adapted to local songs and vocal exercises. Sessions included relaxation and physical awareness exercises, physical warm-up, breathing exercises, song repertoire selected collaboratively with participants, and warm-down relaxation. Dance sessions were led by the lead physiotherapist for PR at the MLI who regularly integrates dance movements into his rehabilitation sessions, and KEJP who has developed and run dance sessions for people with long-term conditions. Sessions included a warm-up using simple rhythmic stepping, progressively demanding dance movements, selected and created collaboratively with participants, followed by a warm-down and gentle stretching. The intensity of the sessions was continually adjusted to participants perceived exertion levels. Sample sessions lasted between 20 and 40 minutes and took place in the MLI, in a large room normally used for the exercise component of PR sessions.

For peer review only

Data collection

Semi-structured interviews were conducted in October 2019, in the MLI, in private rooms, with no non-participants present. The topic guide was developed by reviewing conceptually related research projects conducted by the team and others (see supplementary file 1 'Topic Guide'). Interviews focused on open-ended questions, with participant prompts to encourage further discussion on topics which appeared meaningful. Interviews were conducted by KP, LC and GN, in English or Luganda (predominant local language) depending on participant preference. If in Luganda, GN, an experienced qualitative researcher, translated simultaneously. Interviews were audio-recorded, and interviewers documented immediate reflections following interviews. Interviewer participant relationships were established through relaxed introduction, and participants were informed the interviewers were health professionals, but not directly involved in the provision of their individual healthcare. Modified Medical Research Council (mMRC) breathlessness scores were self-rated by patient participants using the mMRC scale, with options read out loud by the interviewer.

Structured observations of trial singing and dance sessions (see below) were conducted by KP, LC and GN (see Supplementary file 2: Structured observation proforma), and relevant documents analysed (Supplementary file 3: Preparatory Reference Materials), to support contextualisation and interpretation of interview data.

Daily meetings took place involving (depending on availability) GN, IK, KP, LC, RJ, BK, and WK, (DW from the UK) during which ongoing data collection and interpretation was discussed and triangulated with interview notes, structured observations and preparatory reference materials. This process aimed to facilitate understanding and inform the iterative development of ongoing data collection activities.

The participants were informed of the intention and focus of the research, and that their responses in no way influenced their ongoing care, rather that the intention was to inform the development of future interventions, if appropriate. Data were collected and handled as per COnsolidated criteria for REporting Qualitative studies (COREQ)-guidelines[23].

Data analysis

Interviews were transcribed verbatim. KP, LC and DW conducted a thematic analysis based on that described by Braun and Clarke[24] and Terry et al[25]. During phase 1, transcripts were read and re-read, with further listening and familiarising with interview recordings, interviewer reflections, and structured observations. Importantly, notes from discussions between GN, IK, KP, LC, RJ, BK, and WK made during data collection where used to facilitate understanding. Phase 2 included open free-coding, discussion, double-coding, cross-case analysis, and development of coding structure. As such the analysis was predominantly inductive in nature, though deductive elements were contributed by the semi-structured nature of using a topic guide. The coding structure was then refined into preliminary themes (phase 3), which were further discussed, refined, named, and agreed upon (phases 4 and 5). Participant validation was performed with staff members at the MLI. Given current COVID-19 restrictions, further patient participant validation was not performed, however, the clarity and inter-participant consistency of identified themes suggests that further participant validation would have been unlikely to dramatically alter findings. Theme saturation was achieved during the analysis, however given the exploratory nature of the study, all data were analysed. Coding and theme development used Microsoft Excel. Demographic and disease-specific information was sought from

For peer review only

patient participants. Breathlessness scores were completed as an indication of disease-related functional impairment. This was selected given the heterogeneity of lung conditions represented, hence a generic rather than disease-specific assessment was appropriate. Additionally, breathlessness is a key assessment criterion for PR, hence relevant for the application of this study's findings.

Patient and Public involvement

Participant feedback collected during the 'Singing for Breathing Uganda' project evaluation, combined with consultation with patients attending respiratory clinics, prompted this study and informed the topic guide development. Additionally, the primary objective of the study is an exploration of patient and healthcare provider perspectives, hence patient and public involvement is at the core of this study.

Ethical approvals and consent

Ethical approval was granted by the Mulago Hospital Research and Ethics Committee (Reference number MHREC:1478) and the University of Plymouth Faculty Research Ethics and Integrity Committee (19/20-1164). All participants provided written informed consent. All research activities were conducted in accordance with the principles of the Declaration of Helsinki.

Results

19 participants were included in the study, made up of eleven patients and eight staff members who were approached and recruited. Two further patients were approached and declined, stating they did not have time. Regarding the patient participants, eight of the eleven were female, mean age 43 years (range 20-63). Regarding ethnicity, all participants were black Ugandan. All patient participants reported CRD, including PTBLD (x6), post 'infection' lung disease (x1), asthma (x2), COPD (x1), pulmonary fibrosis (x1). mMRC scores ranged from 1 to 3 (mean 1.5). None of the patients used ambulatory oxygen. Various symptoms were reported by patient participants, in keeping with their CRD, including breathlessness, cough, and physical activity limitations. All reported living in houses (rather than flats or 'other'). Two lived alone, nine were cohabiting with family. Seven were in paid employment, one was a student, and three were unemployed. In order to gain the perspectives of individuals with varying amounts of exposure to these kind of interventions, two patients were interviewed prior to sample sessions, while the other nine were interviewed after attending sample sessions, and two of these had also attended SFB Uganda the previous year.

Regarding the eight healthcare professionals, four were women, four men, mean age 41 years (range 29-59). Occupations represented were physiotherapist, respiratory researcher, administrator, carer (sister of a patient), nurse, and three doctors. Participant quotes below are preceded by a 'P' for patient, or 'S' for staff, and the participant number.

On most topics, perspectives between patients and healthcare professionals aligned closely. Our analysis identified four key themes: Music and dance as 1) central components of daily life; having an 2) Established role supporting health and wellbeing; and perceived as having 3) Strong therapeutic potential in respiratory conditions. However, the potential realisation of this 'strong potential' (theme 3)) was dependent upon theme 4) Modulating demographic considerations of cultural and religion, and age.

For peer review only

Theme 1: Music and dance as central components in daily life

Music and dance were described as omnipresent in the social, religious and cultural components of daily life in Uganda. Music and dance were largely inseparable from one another, and described as inclusive and participatory

> S1 *'music really is everywhere for us…Music is really part of our fabric as a society….when they play a song everyone identifies to and everyone is getting up and just dancing, it doesn't matter whether they're in a suit, they're jumping, dancing.'*
>
> and
>
> P4 '*my wife is a politician, when we go to rallies, they normally invite you to come and join them. We join them. Yes. We join them and dance.'*

Music's omnipresence was attributed to its multiple social functions, especially forging interpersonal connectivity

> S1 *'dancing is a way of communing, of interacting with people. It is one of those things that bind people.'*, and P3 *'There is that kind of relationship, with people you sing with.'*
>
> and
>
> S3 *'music speaks to our situations or just that feeling of being together with people and you're singing and you're dancing.'*

A further function being information transfer

> P11 *'Music is very important in our society because it gives messages, it educates through music you are able to know what is good, what is bad, what can be done, what happened in the past, what will happen in the future, all can be delivered through music'*

Participation in music and dance was generally referred to free-willed choice, however many also described a compulsion, as if driven by an external '*power of music*' that overcomes inhibiting factors

> P9 '*[I] feel the music in [me] and [have] to dance*'.

Theme 2: Music and dance had an established role supporting health and wellbeing

Through their role in social, religious, and cultural aspects of life, music and dance were seen as already having established roles in supporting physical, mental, and social health. Such effects were often described as concurrent and interrelated.

Mental health

The most dominant established health-promoting roles related to mental health. Most patients identified this function

> P3 *'[listening to music] you feel happy, you feel you are getting connected with the world that you are not seeing. If gives you some hopeful times. It gives a message. I keep with the message that gives some hope for the future.'*

For peer review only

And

P9 'instead of getting angry, [I] would try and find comfort in singing and dancing to control [my] anger'

Healthcare professionals also highlighted these functions, for patients, but also frequently described using music for stress relief and relaxation themselves. Psychological benefits were underpinned by enjoyment of participation

P8 'I feel nice when I'm singing'

and

S8 'When we are singing, of course you feel like you ... you feel that joy'

Physical health

Physical health improvements were mainly attributed to dance or exercise to music

S4 *'now [dance] has been taken up as one of the things that's used for physical exercises.'*

A group of doctors had also started an afterwork exercise group where they use music for working out, with dance often seen as preferential to other forms of physical activity

S4 'I don't like walking, if I have a car, I will drive it. Even to the nearest distance. But I would do dancing as a physical activity and I would do it with love. Because I love it and I love music.'

Compared with dance, purely physical health benefits were not frequently attributed to singing in its established (daily life) roles, however potential physical benefits of singing were mentioned in relation to singing used in a therapeutic context with patients (see Theme 3).

Social benefits

Social connectivity, as described in theme 1, supported social health and overlapped with mental health and wellbeing.

P4 *'you are joining other people. You know, when you are a people orientated person, when you find people that are happy, you also become happy.'*

This was unpinned by the light-hearted enjoyable nature of music and dance participation

S1 *'it's a fun activity. It's a fun bonding activity for us. Everyone dances whatever they have, silly strokes, and you're just laughing and having a good time.'*

Theme 3: Music and dance perceived as having strong therapeutic potential in respiratory disease management

Contextually appropriate

For peer review only

Perceived potential for successful integration was clear, largely due to the ubiquity (theme 1) and established roles (theme 2) of music and dance in promoting health and wellbeing

> S3 *'because of what our culture is we love partying, we love music, we love dancing, so I think if someone who is told that if you dance, if you sing it is going to improve your health I believe they will have no problem taking part of it.'*

Again, fundamental to the perceived potential, was enjoyment, and group participation

> P1 *'I think it's good to do it as a group. Because you encourage each other. I think it's also more fun, yeah, and then it makes it, you know, something which you've got faster, you move on longer.'*

Potential psychosocial impacts for patients' health conditions were highlighted

> S1 *'no amount of medicine can give you that human connection, which is a very important part of management.'*

Potential therapeutic mechanisms for physical improvements was also suggested by healthcare professionals

> S7 *'you go beyond your tidal volume, in terms of reaching out your respiratory effort … if they keep doing this song then every other time they have some incremental effort required of their respiratory muscles.'*
>
> and
>
> S8 *'I feel it helps because it requires breath control, breathing in, breathing out and at the end it is fun… And of course they are learning also how to sing, how to control their breath, which in their own way helps their healing process and of course coping with the environment.'*

The potential for delivery with minimal resource requirements was emphasised as an important factor, particularly where resources were most limited.

Health benefits

Comments regarding potential health benefits for CRD patients related closely to the established roles of music and dance in wider society.

Physical benefits related to potential exercise training effects, which were seen as very important for people with CRD

> P3 *'with the singing, you feel the lungs, you know, get opened, you feel you breathe very well. You feel the body also, the body moves with the singing, and also dancing. It becomes more free.'*

Some participants in the sample sessions reported improvements in symptoms, though it is important to highlight that these are subjective reports, and no objective assessment of impacts took place.

> P3 '*the sputum can come out very easily*.'
>
> And,

For peer review only

S5 *'That their breathlessness has reduced so they can work a bit longer than they used to. Most of them, that's what they are saying.'*

Improvements in physical symptoms were intimately linked to psychological impacts

P2 *'I was feeling a bit happier because I feel like I could breathe a bit better.'*

The role of social aspects within the sample sessions were noted as creating peer support,

S6 *'It gives them courage and also helps them for the rehabilitation that they're supposed to do. Friends encourage each other to exercise. So it ends up being very, very efficient for them.'*

Enjoyment

Sample session participants were very positive about the experience, which was also noted by staff

P6 *'People were excited, and they say that let us do this whenever we come. They have been so touched. At first we thought, what is this now? But at the end, it has been perfect.'*

As in theme 1, enjoyment was a facilitator of health impacts, and the novelty of the approach was noted positively. Additionally, as in theme 2, participants emphasised the need to adapt sessions to the specific participants of a session (see theme 4).

Already happening

The lead physiotherapist for pulmonary rehabilitation was already integrating dance into his sessions and reporting very positive responses

S2 *'when you bring in a warm-up that is full of dancing and rhythmical, we see they are happy.'*

Also, one patient reported using music for disease specific self-management

P4 *'when I get attacks, I go in my room, and what do I put on? The radio. So, what am I doing? Listening to music.'*

Theme 4: Modulating demographic considerations

Participants emphasised that, for successful implementation, activities or interventions would need to be adapted to the specific participants of any one group, and the group itself. Key factors for consideration to ensure appropriate content included culture and religion, age, gender, and extent of urbanisation. These factors were important for two reasons. Firstly, to ensure that no member of the group felt uncomfortable or excluded. Secondly, responsive contextualisation was seen as a tool to optimise engagement and enjoyment – by selecting songs, music or dances that had cultural or historical significance for the group, a sense of collective identity could be established. This would facilitate interpersonal interactions based on shared experience and knowledge. Such an approach was almost presented as being obvious by participants, as this was how music and dance are used in Uganda more broadly. Contextualisation and personalisation were seen as being part of the essence of music and dance themselves

For peer review only

S2 *'dancing has no formula, it has no pattern. It's not a matter of, oh you must conform. Each one has their own dance. I believe that if I was dancing with you, you have your own style of dancing, and I have my own style of dancing.'*

Culture and religion

For the study respondents, the concepts of culture and religion were interrelated. The terms 'culture' or 'traditional' were often used in reference to traditional tribal practices, beliefs and identities, while 'religion' referred to world religions (Christian, Muslim, or atheist/agnostic)

S2 '*those folk songs, traditional, that people can engage to traditional dances that train from tribe to tribe.*'

Culture is extremely important in Uganda, and music and dance are core to these aspects of daily life (Theme 1)

P1 '*There is no culture in Uganda where there isn't dancing.*'

However, expressions and norms differ

S1 *'every culture, every part of this country has a different kind of dance.'*

Similarly, religion is very important. In Kampala the majority of people identify as Christian, of various denominations, with a smaller but significant proportion follow Islam (14%)[26]. Music and dance are prominent in religious practices and contexts

S8 *'We rarely go direct into praising, praying without singing, without dancing... of course giving glory to god, giving your leg, you are giving your arms, so why not dance.'*

and

P6 *'for the Christians, they are used to singing, because in churches, Protestants do sing. Catholics do sing. Adventists do sing. Born again, most of the people... even the Witch crafts they have their praise, they praise. Yeah. People are used to singing. And Muslims sometimes they do sing.'*

It was suggested that Muslim participants might find singing and dance less acceptable, however, the one Muslim participant was positive about the sample session

P9 *'the dancing helps [me] so much, it's so uplifting.'*

Cultural norms were also highlighted such as issues around exposing parts of the body in close proximity, or how social status may influence acceptability and participation

S7 *'"I'm a Sheikh. I'm a Bishop. I'm a very tough father at home." You know, that kind of person who has a very cut-out social role they probably won't come to sing so much... Such a patient might think that singing might be lowering their social role.'*

Differences between urban and rural norms were highlighted

P1 *'in the rural areas dancing is more associated to ceremony party, not a day to day.'* P1 rural areas are more conservative *'dresses that are longer, skirts like longer, no slits.'*

For peer review only

Age

As per Theme 1, music and dance were described as having multiple functions, the predominant function for an individual was seen to be modulated by age

> S7 *'the old people they still love their music. Where it's a story telling song or it's something to harmonise and move or to advance excitement at a party. Yeah generally the young people of course they love it. Dancing and shaking around.'*

However, age was not seen as a barrier

> S7 *'[older people] like dancing, and quite many of the old they get excited and dance.'*

The perception of others

The importance of these demographic factors also related to how participation might be seen by others, including family, friends and the wider community. Overall, if the activities were clearly being delivered in a therapeutic capacity, participants felt that social acceptance would be high

> P8 '*[my family members] are excited, they want the results afterwards.*'

Improving acceptability

Given these considerations, participants suggested various ways to optimise acceptability. An emphasis on dance being physical exercise was proposed. Additionally, clearly stating the intended therapeutic benefits was important. Similarly, the therapeutic intention of the singing was important, and this was well communicated during the sample sessions

> P8 *'The singing, it is a different kind of singing also, yes, not all songs. But just get songs that push the lungs, expands the lungs, makes the lungs okay, yeah. And the dancing, it depends on the strokes you make, there are dance strokes that stretches the muscles.'*

Health professionals felt acceptance would be more forthcoming if a clear evidence base was also provided. And using the local languages was described by one participant as a method of increasing engagement through cultural identification

> S7 '*they will be more interested in the songs which are done in the local languages. They are richer in terms of connection with the audience.*'

Appropriate song selection would be facilitated by using secular music and co-creating session content specific to the group. This approach worked well in the sample sessions

> P6 '*we sang our national anthem of Uganda. It is for all of us.*'

Of note, although the demographic variables highlighted were considered important by study participants, they were eclectic in their music preferences, with culture and religious norms seen as informing, rather than limiting

> S2 *'The trend is from cultural, traditional, to any pattern somebody wishes to.'*

Additional implementation factors

For peer review only

In addition to demographic considerations, there was a broad appreciation that session content would also be adapted to the physical capacity of individual participants. Participants in the sample sessions felt such adaption took place successfully

> P1 *'for those who are a bit weak, to know that they can rest, when the body feels that it is tired. I thought that that was good.'*

Also, financial and time costs would need consideration to facilitate attendance. Suggestions included having sessions a maximum of once weekly, and subsidising travel costs, to ensure sessions led to net benefit rather than risk contributing to already strained financial situations.

Discussion

The results of this in-depth qualitative study show that music and dance are core components of daily life in Uganda. Study participants felt that participation supports both collective and individual health and wellbeing. These functions supported the perspective, from patients and healthcare professionals, that music and dance had great potential to improve elements of physical, mental and social health and wellbeing, for people with CRD. Individuals who had prior experience of arts-in-health activities, or who participated in sample sessions, were very enthusiastic about the concept. Those without prior experience could see value in the concept and were happy to try. They highlighted important factors for consideration for co-development and successful implementation primarily related to culture and religion, and age.

This study has multiple strengths. Firstly, to our knowledge it is the first to explore this topic. Secondly, the wide-ranging expertise of the research team strengthened interpretation. Thirdly, using in-depth interviews, triangulated with structured observations and key documentation, enabled a detailed, highly contextualised exploration of themes. Fourthly, purposeful convenience sampling ensured appropriate representation from relevant stakeholders.

Certain study limitations and considerations are important to discuss. Firstly, being a single site study, with a sample of 19 participants, the transferability of findings cannot be ascertained, particularly regarding areas of Uganda outside of Kampala, where social and cultural groups and norms are likely to differ. However, Kampala is a district that has a mixture of all tribes in Uganda, and the MLI is a specialist centre, receiving referrals from all over the country. Secondly, COVID-19 pandemic restrictions relating to certain group activities, including singing and exercise[27] are currently in place, and these data were collected prior to the pandemic, hence, the COVID-19 related concerns may change the experience of group activities such as music and dance when they are considered safe to recommence. Additionally, here we report participants' perceptions regarding potential health benefits, and although in general, beneficial effects of similar interventions have been demonstrated, formal research and evaluation of this specific intervention is still required.

Although no other studies have investigated this topic in low-resource settings, our findings echo those of research in related contexts. Research on Singing for Lung Health (SLH) in the UK suggests participants perceive a range of physical, psychological and social benefits in keeping with our findings[11 14 19 21 28 29]. Similarly, studies regarding the perceived impacts of dance for people with long-term respiratory conditions in the UK and Canada identify a range of biopsychosocial benefits[10 12 30]. Additionally, an evaluation of SFB Uganda, a singing project for people with CRD in Uganda, provided anecdotal reports that participation was enjoyed[22], and our findings are broadly in keeping with the evaluation of SFB. Similarly, anecdotal experience of related singing and dance projects for people

For peer review only

with long-term respiratory conditions in other low-resource settings have been enjoyed with participants reporting a range of biopsychosocial benefits[31]. A study of culturally adapted PR in the MLI also showed high-levels of acceptability[8]. Importantly, in each situation described, contextual adaptation and co-development of activities appears crucial to success. Interestingly, there was close alignment regarding responses from patients and healthcare professionals. This may be expected regarding the general role of music and dance in Uganda but was also the case in relation to potential therapeutic interventions. The main differences between the groups were healthcare professionals discussing potential therapeutic mechanisms in more depth and emphasising the requirement for an evidence base to increase acceptance. Future research should include assessing the impact of participation on relevant health outcomes and physiological parameters, building on related physiological research already completed[13].

The current COVID-19 pandemic has necessitated certain restrictions on group activities such as singing[27] and dance, which are likely to impact the potential application of these findings in the short-term. However, developments including widespread immunisations, infection control measures, and remotely delivered singing and dance interventions[11 32 33] may help reduce risk. Additionally, although the majority of participants were highly positive about participation, there were exceptions. As such, music and dance could be used as optional adjuncts to optimise uptake and completion of established, evidence-based respiratory management approaches such as PR.

Conclusions

Long-term respiratory conditions are common in Uganda, causing a high burden of morbidity and mortality. Low-cost, low-resource, interventions are of wide-reaching interest. Our findings suggest people with CRD, and healthcare professionals, see a great deal of potential for the use of music and dance as adjunctive roles to PR, or possibly be delivered as independent activities within CRD management. Building on established therapeutic roles of music and dance in wider Ugandan society, through co-produced intervention development specific to respiratory patients, appears to be a viable route for intervention development. These findings are important for developing arts-in-health interventions in Uganda and beyond.

Information on Researchers:

First Author: KEJP is a 35-year-old male respiratory physician who previously worked as a dancer and dance teacher, with experience leading community dance and dance for people with respiratory disease in the UK, South America, South Africa, Uganda and the Kyrgyz Republic. He lived in Uganda for 8 months during 2015 and 2016, and South Africa for 2 years. KEJP has received training in qualitative research methods from the Imperial College, University College London, and through self-directed learning. He is currently completing a PhD at Imperial College, using qualitative and quantitative methods. Qualifications: MBChB, BSc, MRCP, EADTMH, DPMSA.

DW has extensive experience of qualitative research methods and applied health services research in the UK and international settings and co-designed the research methods for this study. Qualifications MA

For peer review only

Mark William Orme, 30yo, male, senior researcher and research manager for global health project on pulmonary rehabilitation in LMIC including Uganda, PhD. Experience and informal training in qualitative design (interviews and focus groups), conduct (interviews) and analysis (thematic).

Evelyn Brakema, female, 31 years old from the Netherlands, PhD-candidate Global Health (implementation of interventions targeting chronic respiratory disease in Uganda and other low-resource settings) & MD in training for family physician (please decide if relevant). Experience and formal training in qualitative research, particularly from the Horizon 2020 FRESH AIR Project.

Co-authors: LC, DW, GN, IK, BK, EB, MO, DF, NS all have training and experience in qualitative research methods.

Senior author: WK has extensive qualitative and quantitative research experience.

References

1. Ferkol T, Schraufnagel D. The global burden of respiratory disease. *Annals of the American Thoracic Society* 2014;11(3):404-6. doi: 10.1513/AnnalsATS.201311-405PS
2. van Zyl Smit RN, Pai M, Yew WW, et al. Global lung health: the colliding epidemics of tuberculosis, tobacco smoking, HIV and COPD. *The European respiratory journal* 2010;35(1):27-33. doi: 10.1183/09031936.00072909 [published Online First: 2010/01/02]
3. World Health Organisation. GLOBAL STATUS REPORT on noncommunicable diseases 201. Switzerland The WHO, 2014.
4. Salvi S. The silent epidemic of COPD in Africa. *The Lancet Global health* 2015;3(1):e6-7. doi: 10.1016/S2214-109X(14)70359-6 [published Online First: 2014/12/30]
5. van Gemert F, Kirenga B, Chavannes N, et al. Prevalence of chronic obstructive pulmonary disease and associated risk factors in Uganda (FRESH AIR Uganda): a prospective cross-sectional observational study. *The Lancet Global health* 2015;3(1):e44-51. doi: 10.1016/S2214-109X(14)70337-7
6. van Gemert F, van der Molen T, Jones R, et al. The impact of asthma and COPD in sub-Saharan Africa. *Prim Care Respir J* 2011;20(3):240-8. doi: 10.4104/pcrj.2011.00027 [published Online First: 2011/04/22]
7. Jones R, Muyinda H, Nyakoojo G, et al. Does pulmonary rehabilitation alter patients' experiences of living with chronic respiratory disease? A qualitative study. *Int J Chron Obstruct Pulmon Dis* 2018;13:2375-85. doi: 10.2147/COPD.S165623 [published Online First: 2018/08/21]
8. Jones R, Kirenga BJ, Katagira W, et al. A pre-post intervention study of pulmonary rehabilitation for adults with post-tuberculosis lung disease in Uganda. *Int J Chron Obstruct Pulmon Dis* 2017;12:3533-39. doi: 10.2147/COPD.S146659 [published Online First: 2017/12/23]
9. Philip K, Lewis A, Hopkinson NS. Music and dance in chronic lung disease. *Breathe* 2019;15(2):116-20. doi: 10.1183/20734735.0007-2019
10. Wshah A, Butler S, Patterson K, et al. "Let's Boogie": FEASIBILITY OF A DANCE INTERVENTION IN PATIENTS WITH CHRONIC OBSTRUCTIVE PULMONARY DISEASE. *J Cardiopulm Rehabil Prev* 2019;39(5):E14-E19. doi: 10.1097/HCR.0000000000000428 [published Online First: 2019/08/30]
11. Philip KE, Lewis A, Jeffery E, et al. Moving singing for lung health online in response to COVID-19: experience from a randomised controlled trial. *BMJ open respiratory research* 2020;7(1) doi: 10.1136/bmjresp-2020-000737 [published Online First: 2020/11/27]
12. Philip KEJ, Lewis A, Williams S, et al. Dance for people with chronic respiratory disease: a qualitative study. *BMJ open* 2020;10(10):e038719. doi: 10.1136/bmjopen-2020-038719 [published Online First: 2020/10/15]

For peer review only

13. Philip KE, Lewis A, Buttery SC, et al. Physiological demands of singing for lung health compared with treadmill walking. *BMJ open respiratory research* 2021;8(1) doi: 10.1136/bmjresp-2021-000959 [published Online First: 2021/05/29]
14. Kaasgaard M, Rasmussen DB, Ottesen AL, et al. Sing-a-Lung: Group singing as training modality in pulmonary rehabilitation for patients with Chronic Obstructive Pulmonary Disease (COPD): A multicenter, cluster-randomised, non-inferiority controlled trial. *ERJ* 2020;56 doi: 10.1183/13993003.congress-2020.4663
15. Lee AL, Desveaux L, Goldstein RS, et al. Distractive Auditory Stimuli in the Form of Music in Individuals With COPD: A Systematic Review. *Chest* 2015;148(2):417-29. doi: 10.1378/chest.14-2168 [published Online First: 2015/03/06]
16. Fancourt DaF, S.,. What is the evidence on the role of the arts in improving health and well-being? A scoping review (2019): World Health Organisation, 2019.
17. Daykin N, Gray K, McCree M, et al. Creative and credible evaluation for arts, health and well-being: opportunities and challenges of co-production. *Arts & Health* 2017;9:123-38. doi: 10.1080/17533015.2016.1206948
18. Brakema EA, Vermond D, Pinnock H, et al. Implementing lung health interventions in low- and middle-income countries: a FRESH AIR systematic review and meta-synthesis. *The European respiratory journal* 2020;56(1) doi: 10.1183/13993003.00127-2020 [published Online First: 2020/04/29]
19. Lewis A, Cave P, Hopkinson NS. Singing for Lung Health: service evaluation of the British Lung Foundation programme. *Perspect Public Health* 2018;138(4):215-22. doi: 10.1177/1757913918774079 [published Online First: 2018/05/15]
20. Lewis A, Cave P, Hopkinson NS. Singing for Lung Health: a qualitative assessment of a British Lung Foundation programme for group leaders. *BMJ open respiratory research* 2017;4(1):e000216. doi: 10.1136/bmjresp-2017-000216
21. Lewis A, Cave P, Stern M, et al. Singing for Lung Health-a systematic review of the literature and consensus statement. *NPJ primary care respiratory medicine* 2016;26:16080. doi: 10.1038/npjpcrm.2016.80
22. Downes C, Philip KEJ, Lewis A, et al. Singing for Breathing Uganda: Group singing for people with chronic lung disease in Kampala. *Journal of Applied Arts & Health* 2019;10(2):219-28. doi: 10.1386/jaah.10.2.219_1
23. Tong A, Sainsbury P, Craig J. Consolidated criteria for reporting qualitative research (COREQ): a 32-item checklist for interviews and focus groups. *Int J Qual Health Care* 2007;19(6):349-57. doi: 10.1093/intqhc/mzm042 [published Online First: 2007/09/18]
24. Clarke V, Braun, V., Hayfield, N.,. Thematic Analysis. In: Smith JA., ed. Qualitative Psychology: SAGE 2015:222-48.
25. Terry G, Hayfield, N., Clarke, V., Braun, V.,. Thematic Analysis in: The SAGE Handbook of Qualitative Research in Psychology. London: SAGE Publications Ltd 2017:17-36.
26. Uganda Bureau of Statistics. 2014 Census, 2018.
27. Philip KEJ, Lewis A, Buttery SC, et al. Aerosol Transmission of SARS-CoV-2: Inhalation as Well as Exhalation Matters for COVID-19. *American journal of respiratory and critical care medicine* 2021 doi: 10.1164/rccm.202012-4445LE [published Online First: 2021/02/03]
28. Lord VM, Hume VJ, Kelly JL, et al. Singing classes for chronic obstructive pulmonary disease: a randomized controlled trial. *BMC pulmonary medicine* 2012;12:69. doi: 10.1186/1471-2466-12-69
29. Lord VM, Cave P, Hume VJ, et al. Singing teaching as a therapy for chronic respiratory disease--a randomised controlled trial and qualitative evaluation. *BMC pulmonary medicine* 2010;10:41. doi: 10.1186/1471-2466-10-41
30. Harrison SL BK, Edwards J, McFaull V, McLusky S, Russell A, Williams G, Williams S. DANCE FOR PEOPLE WITH CHRONIC BREATHLESSNESS: A FEASIBILITY STUDY. Thorax, 2019.

31. Philip KEJ, Katagira W, Jones R. Dance for Respiratory Patients in Low-Resource Settings. *Jama* 2020;324(10):921-22. doi: 10.1001/jama.2020.15426 [published Online First: 2020/09/09]
32. Philip K, Lewis, A., Harrison, S.,. Singing and Dance for People with Chronic Breathlessness during the COVID-19 pandemic: American Thoracic Society 2020 [Available from: https://www.thoracic.org/members/assemblies/assemblies/pr/quarterly-bite/singing-and-dance-for-people-with-chronic-breathlessness-during-the-covid-19-pandemic.php accessed 29/07/2020 2020.
33. Philip KEJ, Lewis A, Jeffery E, et al. Moving Singing for Lung Health online: experience from a randomised controlled trial. *medRxiv* 2020

For peer review only

Kupumua project – October 2019

Topic guide interviews –family members

1. How much do you listen to music at home/work?
    a. What type of music?
    b. Who do you listen with?
    c. How do you listen to music (probe for devices/access)
    d. How often?
    e. Do you sing along?
    f. How does music make you feel?
2. Tell us about dancing:
    a. How much do you dance/(other term?) – how often?
    b. What type of dancing?
    c. Who with?
    d. How does it feel to dance?
    e. Does your condition affect your dancing in any way?
    f. What do your friends/family think about that?
3. How is music and dance regarded in your family?
    a. And in your community?
    b. Do people think it is important? Why?
4. What do you think about singing and dancing as a way to improve health for people like your family member?
5. What do you think other people in your community/family would think about that?
6. Would their opinion make any difference to whether your family member might take part?

IF RESPONSES ARE POSTIIVE TO QS 4-6 THEN ASK

7. What do you think could be any difficulties for your family member in doing singing or dance to improve health?
8. What would be the things that might help them?

For peer review only - http://bmjopen.bmj.com/site/about/guidelines.xhtml

Kupumua Structured Observation Sheet 1

Trial session:

Date:

Observer:

Location:

People present:

Observation of an active session (could be singing, dancing, PR or PR plus music/dance)

Observation	Session type:
1. Body language	
2. Facial expressions	
3. Speech/expression	
4. Interactions between peers	
5. Interactions with staff	
6. Physical involvement with music, singing, dancing	
7. Disease related behaviour (short of breath, coughing, fatigue, resting periods,	
8. Role within the group. Passive/active. Lead/follow.	
9. Reflexive researcher responses	

For peer review only

For peer review only - http://bmjopen.bmj.com/site/about/guidelines.xhtml

Supplementary Materials

Preparatory Reference Materials

- Does pulmonary rehabilitation alter patients' experiences of living with chronic respiratory disease? A qualitative study Int J Chron Obstruct Pulmon Dis. 2018; 13: 2375–2385. doi: 10.2147/COPD.S165623 https://www.ncbi.nlm.nih.gov/pmc/articles/PMC6087019/
- A development study of pulmonary rehabilitation for patients with chronic lung disease in Uganda https://erj.ersjournals.com/content/48/suppl_60/PA858.abstract
- A qualitative study on the development of pulmonary rehabilitation for patients with chronic lung disease in Kampala, Uganda https://erj.ersjournals.com/content/48/suppl_60/PA3964.abstract
- International research and guidelines on post-tuberculosis chronic lung disorders: a systematic scoping review https://gh.bmj.com/content/3/4/e000745.abstract
- A pre–post intervention study of pulmonary rehabilitation for adults with post-tuberculosis lung disease in Uganda https://www.ncbi.nlm.nih.gov/pmc/articles/PMC5729823/
- Chronic Respiratory Symptoms and Lung Abnormalities Among People With a History of Tuberculosis in Uganda: A National Survey https://academic.oup.com/cid/advance-article-abstract/doi/10.1093/cid/ciy795/5099459
- Beliefs and behaviours towards chronic lung disease - a mixed-method FRESH AIR study https://erj.ersjournals.com/content/50/suppl_61/PA3891.abstract
- Late Breaking Abstract - Health economic burden of asthma/COPD in Uganda, Vietnam, Kyrgyzstan and Greece: FRESH AIR results https://erj.ersjournals.com/content/50/suppl_61/OA2911.abstract
- The silent socioeconomic impact of COPD/asthma in Africa, Asia and Europe – a FRESH AIR study https://erj.ersjournals.com/content/52/suppl_62/PA4215.abstract
- Critical implementation factors to lung-interventions in low-resource-settings – a FRESH AIR systematic review https://erj.ersjournals.com/content/52/suppl_62/PA4214.abstract

For peer review only

COREQ (COnsolidated criteria for REporting Qualitative research) Checklist

A checklist of items that should be included in reports of qualitative research. You must report the page number in your manuscript where you consider each of the items listed in this checklist. If you have not included this information, either revise your manuscript accordingly before submitting or note N/A.

Topic	Item No.	Guide Questions/Description	Reported on Page No.
**Domain 1: Research team and reflexivity**			
Personal characteristics			
Interviewer/facilitator	1	Which author/s conducted the interview or focus group?	
Credentials	2	What were the researcher's credentials? E.g. PhD, MD	
Occupation	3	What was their occupation at the time of the study?	
Gender	4	Was the researcher male or female?	
Experience and training	5	What experience or training did the researcher have?	
Relationship with participants			
Relationship established	6	Was a relationship established prior to study commencement?	
Participant knowledge of the interviewer	7	What did the participants know about the researcher? e.g. personal goals, reasons for doing the research	
Interviewer characteristics	8	What characteristics were reported about the inter viewer/facilitator? e.g. Bias, assumptions, reasons and interests in the research topic	
**Domain 2: Study design**			
Theoretical framework			
Methodological orientation and Theory	9	What methodological orientation was stated to underpin the study? e.g. grounded theory, discourse analysis, ethnography, phenomenology, content analysis	
Participant selection			
Sampling	10	How were participants selected? e.g. purposive, convenience, consecutive, snowball	
Method of approach	11	How were participants approached? e.g. face-to-face, telephone, mail, email	
Sample size	12	How many participants were in the study?	
Non-participation	13	How many people refused to participate or dropped out? Reasons?	
Setting			
Setting of data collection	14	Where was the data collected? e.g. home, clinic, workplace	
Presence of non-participants	15	Was anyone else present besides the participants and researchers?	
Description of sample	16	What are the important characteristics of the sample? e.g. demographic data, date	
Data collection			
Interview guide	17	Were questions, prompts, guides provided by the authors? Was it pilot tested?	
Repeat interviews	18	Were repeat inter views carried out? If yes, how many?	
Audio/visual recording	19	Did the research use audio or visual recording to collect the data?	
Field notes	20	Were field notes made during and/or after the inter view or focus group?	
Duration	21	What was the duration of the inter views or focus group?	
Data saturation	22	Was data saturation discussed?	
Transcripts returned	23	Were transcripts returned to participants for comment and/or	

For peer review only

Topic	Item No.	Guide Questions/Description	Reported on Page No.
		correction?	
**Domain 3: analysis and findings**			
Data analysis			
Number of data coders	24	How many data coders coded the data?	
Description of the coding tree	25	Did authors provide a description of the coding tree?	
Derivation of themes	26	Were themes identified in advance or derived from the data?	
Software	27	What software, if applicable, was used to manage the data?	
Participant checking	28	Did participants provide feedback on the findings?	
Reporting			
Quotations presented	29	Were participant quotations presented to illustrate the themes/findings? Was each quotation identified? e.g. participant number	
Data and findings consistent	30	Was there consistency between the data presented and the findings?	
Clarity of major themes	31	Were major themes clearly presented in the findings?	
Clarity of minor themes	32	Is there a description of diverse cases or discussion of minor themes?	

Developed from: Tong A, Sainsbury P, Craig J. Consolidated criteria for reporting qualitative research (COREQ): a 32-item checklist for interviews and focus groups. *International Journal for Quality in Health Care*. 2007. Volume 19, Number 6: pp. 349 – 357

Once you have completed this checklist, please save a copy and upload it as part of your submission. DO NOT include this checklist as part of the main manuscript document. It must be uploaded as a separate file.

For peer review only
